# An evidence-weighted knowledge framework with path-aware reasoning for personalized lifestyle management in chronic disease

**DOI:** 10.3389/fpubh.2026.1924985

**Published:** 2026-09-24

**Authors:** Hongyu Kang, Xiaowei Xu, Ning Xiao, Li Hou, Qin Li, Xue Feng, Jiao Li

**Affiliations:** 1 School of Medical Technology, Beijing Institute of Technology, Beijing, China; 2 Institute of Medical Information/Library, Chinese Academy of Medical Sciences & Peking Union Medical College, Beijing, China; 3 Healthy Lifestyle Medicine Center, State Key Laboratory of Cardiovascular Disease, Fuwai Hospital, National Center for Cardiovascular Diseases, Chinese Academy of Medical Sciences & Peking Union Medical College, Beijing, China

**Keywords:** chronic disease prevention, evidence-weighted knowledge graph, large language models, personalized lifestyle management, public health

## Abstract

**Background:**

Healthy lifestyle management is essential for chronic disease prevention and long-term care. However, evidence linking lifestyle behaviors, biological mechanisms, and health outcomes is dispersed across diverse biomedical sources. This fragmentation makes it difficult for health professionals to efficiently retrieve and interpret evidence needed to inform personalized lifestyle guidance.

**Methods:**

We constructed an Evidence-Weighted Lifestyle Knowledge Graph (EW-LKG) to organize multidimensional lifestyle evidence by extracting structured triples from over 170,000 PubMed abstracts using schema-based large language model extraction with rule-guided verification. Each relation was assigned a heuristic confidence score based on study design hierarchy and aggregated supporting evidence to facilitate evidence-aware retrieval and reasoning. Path-aware retrieval and multi-hop reasoning were used to identify high-confidence evidence chains that support answer generation by large language models. Performance was evaluated by benchmark question answering across four large language models, external validation on the PubMedQA chronic disease subset, and expert scoring across 5 evaluation dimensions.

**Results:**

We constructed an evidence-weighted, multidimensional lifestyle knowledge graph integrating diet, physical activity, sleep, stress-related, and social-behavioral factors, comprising 156 k entities and 202 k relations. Path-aware question answering improved accuracy across four large language models, with Qwen3-Max increased by 0.184, Llama-4-Maverick increased by 0.159, DeepSeek V3.2-Exp increased by 0.161 and GPT-5.2 increased by 0.159. Correct answers were also associated with higher PathScores (0.80 vs. 0.62), particularly for mechanism-mediated and multi-factor reasoning. Results showed insensitivity across alternative evidence weighting schemes, and source-disjoint validation on the lifestyle-domain benchmark suggested that gains derived from structured knowledge integration rather than corpus overlap; on the general-domain PQA-L split, gains were smaller and narrowed in the source-disjoint subset (0.598 vs. 0.528; 0.464 vs. 0.435). External validation on PubMedQA chronic disease questions yielded consistent performance. Expert evaluations across five dimensions showed an average gain of 4.34 points.

**Conclusion:**

The EW-LKG provides a structured approach for organizing literature-derived lifestyle evidence and selecting evidence-linked reasoning paths. In the evaluated datasets, path-aware retrieval improved QA accuracy across multiple LLMs. As an evidence-organizing framework, it may support more systematic and evidence-informed lifestyle management, while further validation in real-world clinical and public health settings is needed.

## 1 Introduction

Healthy lifestyle management is widely recognized as an essential component of chronic disease prevention and long-term management (1–3). It is also a cornerstone of public health strategies aimed at reducing the burden of chronic diseases and promoting healthy aging. It typically involves comprehensive interventions spanning dietary patterns, physical activity, sleep behaviors, stress management, and social factors (4–6). These dimensions do not operate independently but interact through complex biological and behavioral pathways (7), requiring clinicians to integrate heterogeneous evidence when formulating personalized recommendations (8, 9). However, lifestyle-related evidence is widely dispersed across heterogeneous biomedical sources and represented in diverse formats, including narrative text, structured metadata, and guideline documents, posing challenges for systematic retrieval and integration (10). The absence of structured and computationally integrated evidence frameworks limits efficient retrieval, organization, and traceable synthesis of research findings (11), thereby restricting the development of scalable and personalized lifestyle guidance for public health.

Despite its recognized importance, translating lifestyle research into actionable clinical guidance remains difficult (12). The underlying evidence linking dietary factors, nutrients, physical activity, and other lifestyle behaviors to chronic disease outcomes is not organized in an interoperable manner (13). Evidence is distributed across heterogeneous publications and varies in methodological rigor, population context, and outcome definition. In routine practice, particularly under time constraints, clinicians lack methods to retrieve, compare, and synthesize this heterogeneous evidence while preserving source traceability (14, 15). Consequently, lifestyle guidance often relies on generalized summaries rather than structured, mechanism-informed, and evidence-quantified support tailored to individual patient characteristics.

Recent advances in large language models (LLMs) have demonstrated strong capabilities in processing and summarizing biomedical text (16–18). Prior studies have shown that LLMs can support literature analysis, relation extraction, and clinical note summarization, reducing clinicians’ information burden. A recent scoping review (19) highlights their effectiveness in synthesizing large volumes of biomedical publications, while benchmarking studies (20) show that adapted models can generate clinical summaries comparable to expert-written versions, underscoring their potential to reduce clinicians’ information burden. However, despite their generative fluency, LLM outputs often lack explicit grounding in structured and traceable evidence (21–23). In domains such as lifestyle management, transparency and traceability of generated responses are particularly important. This limitation restricts the use of LLMs as reliable evidence synthesis tools for complex lifestyle-related queries.

Although LLMs have demonstrated strong capabilities in understanding and generating biomedical text, they may still struggle to integrate evidence distributed across multiple concepts and relationships (24). In lifestyle management, the relationship between behaviors and chronic disease outcomes often involves multiple biological and behavioral pathways rather than isolated associations (25). Therefore, structured knowledge paths can help organize related evidence, preserve the connections among concepts, and support more coherent and transparent reasoning.

In this study, we propose an evidence-weighted knowledge representation framework that organizes large-scale lifestyle evidence extracted from biomedical literature and quantitatively represents evidence strength based on study design hierarchy. The framework aims to support evidence retrieval, knowledge organization, and transparent reasoning for personalized lifestyle guidance by structuring and linking heterogeneous lifestyle evidence from biomedical literature. We further introduce a path-aware reasoning mechanism that propagates evidence confidence across multi-hop knowledge graph paths to identify high-confidence evidence chains. This structured reasoning pipeline enables LLMs to generate traceable and evidence-informed responses by connecting distributed lifestyle evidence, facilitating evidence synthesis and public health-oriented lifestyle guidance.

The key contributions of this study are as follows: (i) a structured and evidence-weighted framework for integrating heterogeneous lifestyle evidence from large-scale biomedical literature, with an evidence-informed path ranking mechanism that prioritizes coherent multi-hop reasoning chains. (ii) a path-aware reasoning approach that enables LLMs to generate transparent and evidence-informed lifestyle recommendations for chronic disease management. Together, these contributions provide a practical foundation for personalized lifestyle management while supporting evidence-informed public health strategies for chronic disease prevention.

The remainder of this manuscript is organized as follows. The Related Work section summarizes existing approaches in lifestyle knowledge representation, LLM-augmented biomedical reasoning, and path-aware evidence retrieval. The Methods section describes the construction of the Evidence-Weighted Lifestyle Knowledge Graph (EW-LKG), evidence weighting strategy, path-aware retrieval framework, and evaluation design. The Results section presents graph construction, question answering performance, and application evaluation. The Discussion section further discusses the implications, limitations, and future directions of the proposed framework.

## 2 Related work

### 2.1 Knowledge graphs for lifestyle evidence integration and health management

Knowledge graphs can provide a structured representation of domain knowledge and modeling of relationships among lifestyle factors, biological mechanisms, and disease outcomes (26), facilitating evidence organization for precision public health and personalized prevention. Several domain-specific KGs have been developed to support nutrition and fitness reasoning. For instance, KG4NH (27), a comprehensive graph for dietary nutrition and human health that facilitates structured diet–disease reasoning; Food4healthKG (28) integrated food, gut microbiota, and mental health knowledge for query and recommendation tasks; and, a Chinese fitness knowledge dataset called FitKG-CN (29) covered exercise, nutrient, and physiological concepts across multiple categories. Nevertheless, most existing KGs primarily focus on entity–relation organization and semantic connectivity, while offering limited mechanisms to quantify methodological rigor or integrate evidence hierarchy into downstream reasoning processes.

### 2.2 LLM-augmented knowledge graph reasoning and evidence-aware retrieval

Integrating LLMs with KGs has emerged as a promising strategy to enhance evidence retrieval and reasoning from large-scale biomedical literature (30–33). For example, the MedKA framework (34) integrated a biomedical knowledge graph into LLM training and evaluation to improve factuality in clinical question answering and medical knowledge assessment, aligning model outputs with structured evidence. Similarly, the KRAGEN framework (35) leveraged KG-based retrieval and reasoning in biomedical problem-solving tasks, enabling the decomposition of complex medical queries into substantiated fact chains. Recent approaches have explored evidence-aware modeling in different forms. For example, weighted graph network methods (36) have been proposed to combine conflicting evidence through structured weighting schemes, while multi-evidence RAG frameworks (37) incorporate retrieval confidence and source attribution to enhance factual reliability. However, challenges remain in translating these advances into clinically actionable decision support for lifestyle management.

### 2.3 Path-aware reasoning for biomedical evidence integration

Complex biomedical questions often require integrating information across multiple concepts and relationships, making isolated entity retrieval insufficient for evidence synthesis. Path-aware reasoning has therefore been explored as a strategy to connect related concepts and identify coherent evidence chains in biomedical knowledge systems. Recent studies have demonstrated its potential in improving biomedical discovery and reasoning. Chen et al. (38) investigated the integration of multi-hop medical knowledge into smaller language models for biomedical question answering, demonstrating that structured multi-hop knowledge can improve the utilization of medical information during reasoning. The eGoT framework (39) leveraged biomedical knowledge graphs and multi-hop retrieval to construct connected reasoning chains from biomedical literature, enabling evidence-grounded reasoning and hypothesis generation across interconnected biomedical concepts. These studies suggested that connected evidence chains may provide a more structured basis for interpreting complex biomedical relationships.

Taken together, existing approaches provide valuable foundations for biomedical knowledge organization, LLM-assisted reasoning, and path-based evidence retrieval. However, existing frameworks rarely combine quantitative evidence weighting with path-aware reasoning to organize heterogeneous lifestyle evidence and support traceable personalized lifestyle management.

## 3 Methods

### 3.1 Overview

This study proposes an evidence-weighted framework for structured lifestyle knowledge integration in personalized chronic disease management (Figure 1). Lifestyle-related biomedical literature was systematically extracted under a predefined schema using a large language model and validated through rule-guided consistency checks. The curated, literature-linked triples were incorporated into an Evidence-Weighted Lifestyle Knowledge Graph (EW-LKG), in which each relation was assigned a composite confidence score according to levels of evidence. Building on this quantitative representation, path-aware retrieval was performed to identify high-confidence reasoning paths, followed by multi-hop evidence aggregation with source traceability. The resulting evidence chains were provided to the large language model to generate traceable and evidence-informed lifestyle recommendations, supporting personalized lifestyle management and evidence-informed public health practice.

**Figure 1:**
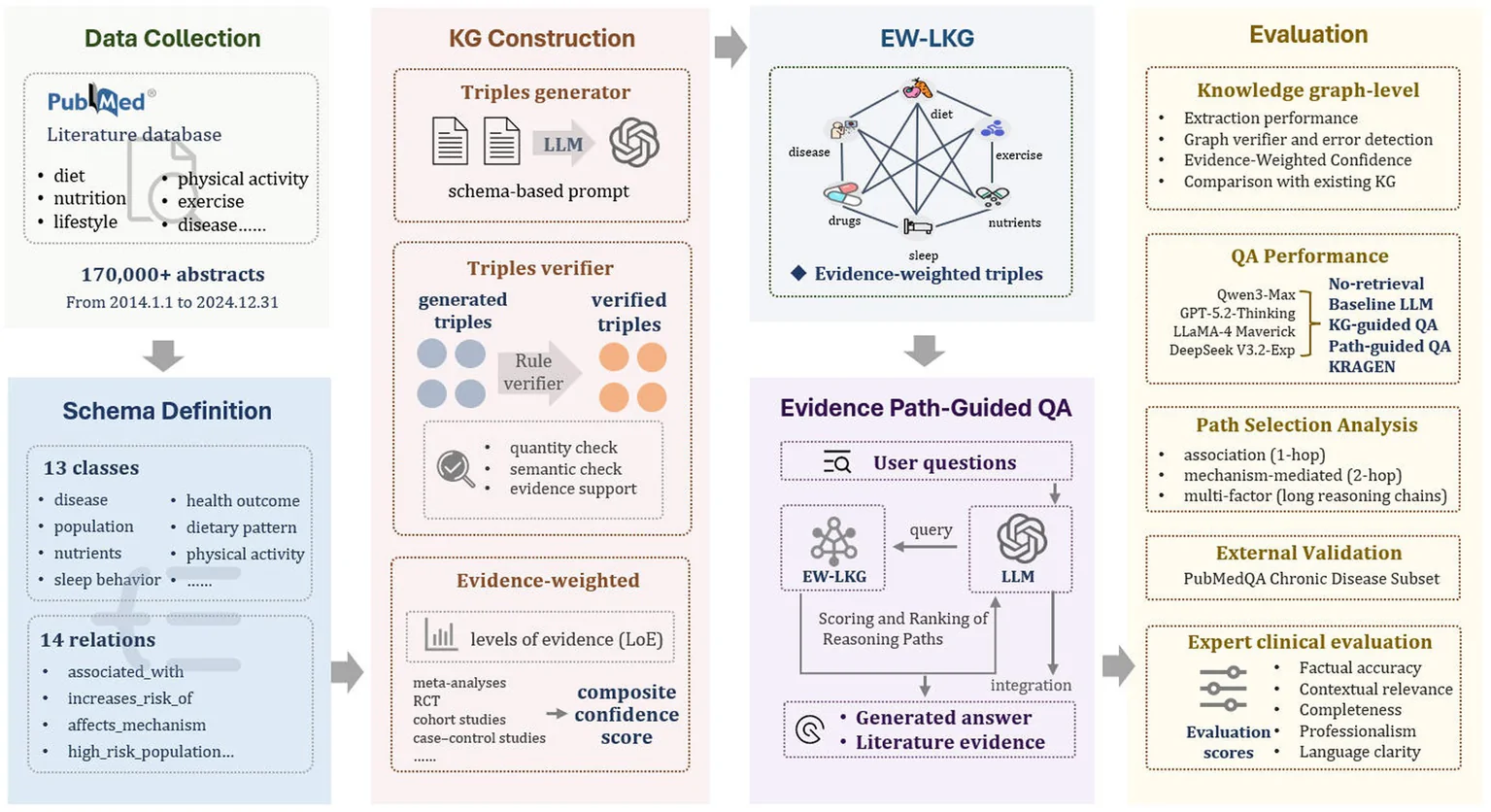
Overview of evidence-weighted knowledge framework. Biomedical literature is extracted and structured into an Evidence-Weighted Lifestyle Knowledge Graph (EW-LKG), followed by path-aware retrieval and reasoning to support traceable, mechanism-informed, and personalized chronic disease management.

### 3.2 Lifestyle knowledge representation

The schema of lifestyle knowledge graph (see schema in Supplementary materials) represented complex interactions between chronic disease lifestyle factors and health outcomes. It was guided by biomedical ontologies (e.g., MeSH), literature (40), guidelines (41, 42), and expert consultation, with the goal of covering the diverse and interacting lifestyle factors relevant to chronic disease prevention and individualized management.

The EW-LKG schema in our study defined 13 entity classes to support evidence-based healthy lifestyle management in chronic disease. It included core entities describing disease processes and outcomes (Disease, Health Outcome, Intermediate Mechanism, and Clinical Manifestation), which captured chronic conditions, risk-related endpoints, and underlying biological pathways. To represent the multidimensional nature of lifestyle management, the schema defined lifestyle-related entities, including Dietary Pattern, Nutrient, Physical Activity, Sleep Behavior, Stress & Mental State, and Social & Behavioral Context, enabling representation of diverse lifestyle factors. In addition, clinical and management-related entities (Population, Intervention, and Drug) were incorporated to contextualize lifestyle evidence to structurally support personalized and population-specific lifestyle recommendations.

The schema also specified 14 relationship types to capture associations, risk-modifying effects, and evidence-supported pathways among entities. These relationship types were designed based on the characteristics of lifestyle-related evidence and the requirements of chronic disease management, where health outcomes are often influenced by multiple interacting behavioral, biological, and contextual factors. Therefore, the schema was developed to represent different levels of evidence relationships, including direct associations between lifestyle factors and health outcomes, intermediate mechanisms linking exposures to outcomes, and population- or intervention-specific factors relevant to personalized guidance. These included relationships linking lifestyle factors to diseases and health outcomes (e.g., increases_risk_of, reduces_risk_of), lifestyle-to-mechanism connections (affects_mechanism, mediated_by), and mechanism-to-disease pathways (contributes_to). Population-specific relevance and intervention effects were represented through relationships such as high_risk_population and recommended_for. Together, these relationships enabled structured reasoning over multifactorial lifestyle evidence and supported evidence-informed information retrieval.

### 3.3 Data collection

Under the predefined schema, we first constructed a MeSH-based concept vocabulary to support consistent literature retrieval. This vocabulary included 1,015 terms: 423 lifestyle-related, 182 disease and health outcome concepts and 410 biomedical entity concepts. Clinical experts in chronic disease management and lifestyle reviewed the vocabulary. The chronic disease scope considered in this study included major lifestyle-associated conditions, such as cardiovascular diseases, type 2 diabetes, obesity, and metabolic disorders. Using this vocabulary, we performed literature retrieval in PubMed by combining concepts across different dimensions, with lifestyle-related terms serving as the primary anchors. PubMed abstracts were selected as the primary evidence source because they provide a large-scale and standardized representation of biomedical research, enabling systematic extraction of lifestyle-related evidence across diverse disease domains.

Publications published from January 2014 to December 2024 were considered. The retrieval yielded 373,566 PubMed records. After duplicate removal (*n* = 2,050), the remaining records were screened to exclude pre-2014 publications, animal studies, narrative or systematic reviews, and irrelevant or insufficient abstracts, leaving 170,268 records for downstream knowledge extraction. Ambiguous cases were manually reviewed to retain human-focused and lifestyle-related evidence. A detailed corpus-selection flow diagram is provided in Figure 2.

**Figure 2:**
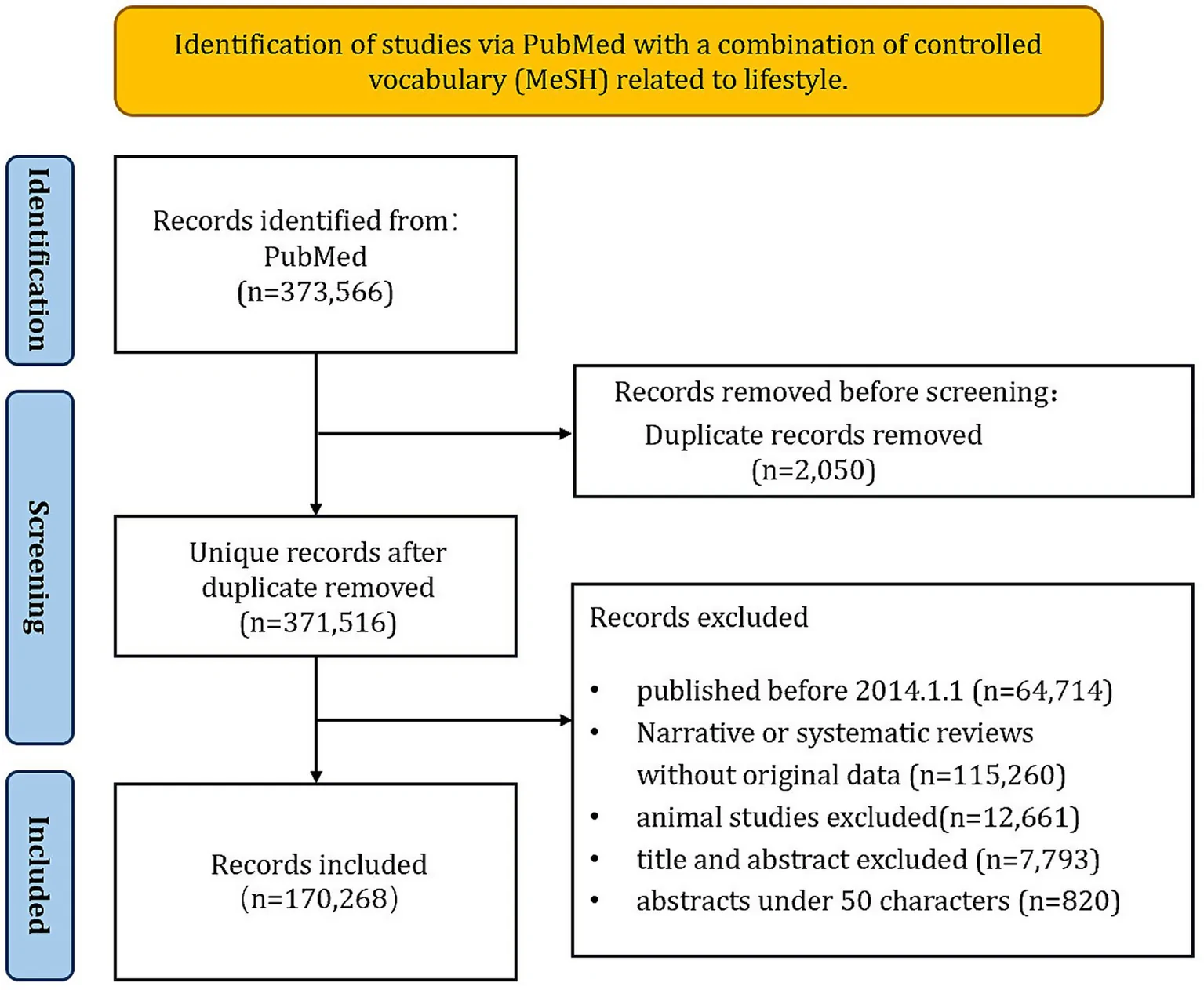
Corpus-selection flow diagram.

### 3.4 Lifestyle knowledge extraction

#### 3.4.1 Structural extraction of lifestyle evidence

We designed a schema-based prompt to support large language model–based extraction of lifestyle-related knowledge from biomedical literature. This prompt specified the extraction task, predefined entity and relation types, and annotation guidelines, thereby guiding the model to identify lifestyle-relevant entities and their semantic relationships in a consistent manner. Representative examples were also included in the prompt to enhance the model’s understanding of lifestyle-related relationships. All extracted results were formatted as structured JSON records, facilitating subsequent verification and integration into the knowledge graph. During information extraction, DeepSeek V3.2-Exp (43) was selected as the underlying language model due to its strong general reasoning capability, biomedical text processing performance, and efficient inference characteristics for large-scale literature analysis. The extraction process was constrained by the predefined schema and structured output format to improve consistency and reduce unconstrained generation. Model parameters were set to a temperature of 0.7 and a maximum token length of 2048. T = 0.7 was chosen to balance precision and recall during relation extraction from linguistically variable biomedical abstracts. Three repeat runs at T = 0.7 showed similar performance (χ^2^ = 0.066, df = 2, *p* = 0.967), with precisions of 0.882, 0.878, and 0.879; recalls of 0.900, 0.885, and 0.906; and F1 scores of 0.891, 0.882, and 0.893. A supplementary extraction run at T = 0 on the same 200-abstract set yielded lower recall (88.2%). The detailed prompts are available in Supplementary materials.

#### 3.4.2 Quality control and evidence validation

To further improve the reliability of knowledge extraction, we introduced a Knowledge Graph Verifier [powered by GPT-5.2-Thinking (44)] to detect and correct erroneous triples identified in the extracted output. The verifier is a rule-guided LLM verification module that applies prompt-based checks. The design of these criteria was informed by general knowledge graph validation principles reported in RuleHub (45), particularly schema and logical consistency checking, and was further extended to address the characteristics of evidence-based lifestyle knowledge. Specifically, the verifier evaluates three aspects of extracted triples: extraction completeness, schema compatibility and evidence support.

During quality control, each extracted triple is sequentially evaluated using these criteria. If a potential error is detected, the corresponding error type is recorded and used to guide the subsequent LLM-based correction process. The verification process consists of sequential error detection and correction, producing more accurate and evidence-consistent triples for downstream graph construction. The detailed prompts are available in Figure 3 and Supplementary materials.

**Figure 3:**
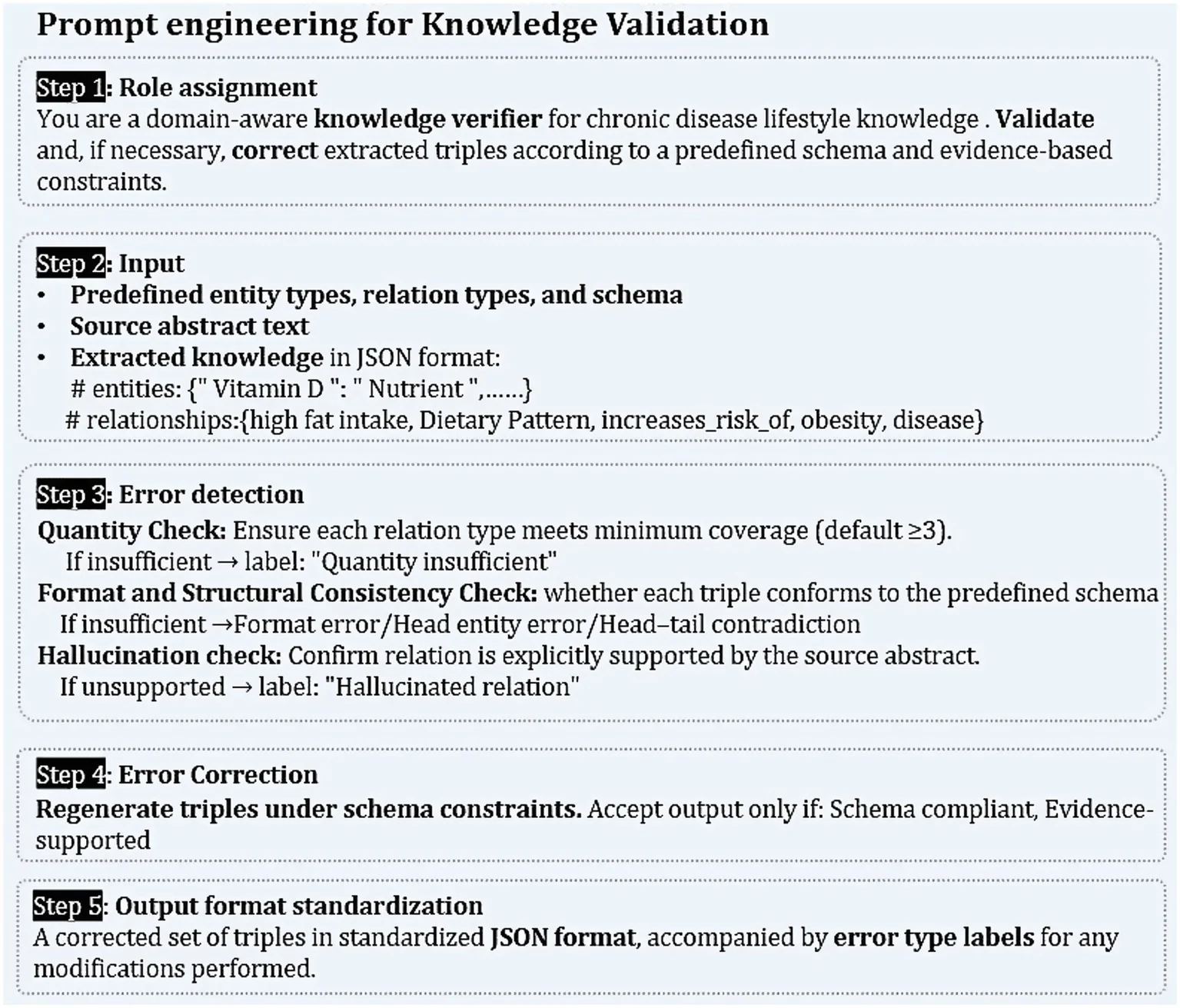
Structured LLM-based knowledge validation prompt. A stepwise prompt was designed for error detection and error correction.

Error detection was conducted in three sequential steps:

- Quantity check

The verifier assesses whether enough triples have been generated. If the number of triples falls below a predefined threshold (default = 3), the output is labeled as quantity insufficient, indicating limited relational coverage that may undermine reliable knowledge integration.

- Semantic consistency check

A generated triple is classified as a semantic inconsistency if it violates schema-defined constraints. This includes cases where an entity is assigned an incompatible predefined type, or where the relation type is not semantically compatible with the types of its head and tail entities according to the schema.

- Evidence support check

LLM-based extraction may generate unsupported relationships that are not explicitly described in the source literature. Therefore, an evidence support check was implemented through a verification prompt, where the extracted triple and corresponding source abstract were provided to the verifier LLM to assess evidence support. Relations lacking sufficient textual evidence were removed or corrected during the subsequent correction process.

Error correction was conducted by re-prompting the LLM with instructions tailored to the detected error type. For example, when a semantic inconsistency was identified, the model was instructed to regenerate the triple in strict accordance with the predefined schema. Multiple correction iterations were permitted and repeated until no further errors were detected by the verifier, after which the final output was retained.

#### 3.4.3 Entities standardization

To improve graph connectivity and reduce entity fragmentation, an entity normalization step was performed prior to graph construction. Extracted entities were standardized and aligned using the MeSH vocabulary as the reference terminology to ensure consistent representation of biomedical concepts across the knowledge graph.

### 3.5 Evidence-weighted lifestyle knowledge graph construction

To improve the reliability of literature-derived relations, we extended our knowledge graph to an evidence-weighted lifestyle knowledge graph. In this extension, each relation was assigned a quantitative confidence score based on publication metadata and the accumulation of supporting studies.

#### 3.5.1 Evidence weighting based on study design

PubMed publication type metadata provides standardized classifications of study design, allowing approximate comparison of methodological rigor across different study types. For each extracted triple linked to PubMed abstracts, the publication type was retrieved from structured metadata to identify the underlying study design. Study designs were then mapped to levels of evidence (LoE) (46), a commonly used framework organizing evidence according to methodological hierarchy, and categorized according to the Oxford Centre for Evidence-Based Medicine (OCEBM) (47) hierarchy. Study types with stronger causal inference, such as randomized controlled trials, were assigned higher weights, whereas observational studies received lower weights (Table 1). The resulting evidence levels were subsequently normalized to a continuous scale ranging from 0 to 1 to facilitate quantitative reasoning.

**Table 1:** Mapping of study designs to evidence confidence weights.

PubMed publication type	Study design category	Evidence level	Normalized weight
Meta-analysis	Evidence synthesis	Level 1 (very high)	0.95
Randomized Controlled Trial	Interventional study	Level 2 (high)	0.90
Cohort Study	Observational longitudinal study	Level 3 (moderate)	0.75
Case–Control Study	Observational analytical study	Level 4 (moderate–low)	0.60
Cross-Sectional Study	Observational descriptive study	Level 5 (low)	0.45
Case Report / Expert Opinion/ others	Descriptive / non-empirical evidence	Level 6 (very low)	0.30

The assigned normalized weights should be interpreted as heuristic coefficients rather than formal evidence quality scores. Factors such as sample size, risk of bias, statistical significance, and effect size were not explicitly incorporated, as rigorous evidence appraisal requires dedicated systematic evaluation. Instead, to support quantitative reasoning while preserving the established evidence hierarchy, normalized weights were assigned according to the ordinal levels in OCEBM framework. The resulting weighting scheme was reviewed by clinicians within the research team to ensure its clinical plausibility and consistency with evidence-based medicine principles.

#### 3.5.2 Aggregation of supporting evidence

In many cases, a single relation was supported by multiple independent studies. To reflect the combined contribution of these studies, we aggregated their individual weights into a composite confidence score that captures the number of supporting sources. Each supporting publication was treated as an independent source during aggregation. The resulting confidence score was designed to provide a relative measure of cumulative evidence support for graph reasoning and relation prioritization, rather than a formal assessment of evidence quality.

$$\text{Confidence}(\text{triple})=1-\prod_{i=1}^{n}(1-w_{i})$$

Where $w_{i}$ denotes the weight assigned to the i -th supporting study. The resulting value ranges from 0 to 1 and provides a relative confidence indicator for comparing relations within the constructed knowledge graph. It combines the predefined methodological weights of supporting studies and the number of available supporting publications to support evidence-aware retrieval and path selection.

Then, triples were imported into the Neo4j graph database to construct EW-LKG. It preserves the original entity–relation structure while enriching each triple with a calibrated confidence attribute. This confidence score was subsequently incorporated into relation ranking and path selection during graph-based retrieval, thereby enabling evidence-aware reasoning in downstream query processing by LLMs.

### 3.6 Path-aware reasoning

To support downstream query answering, we retained an RAG framework in which user queries were translated into Cypher templates to retrieve structured knowledge from the EW-LKG. Instead of returning isolated triples, we introduced a graph-based reasoning path selection strategy.

#### 3.6.1 Identification of candidate reasoning paths

For a given query, relevant source and target entities were identified. Paths were enumerated within a limited hop depth to maintain interpretability and avoid overly long reasoning chains (maximum three hops). For example, when evaluating the association between sugar intake and metabolic syndrome, the system retrieved both direct relations (e.g., sugar → increases_risk_of → metabolic_syndrome) and indirect paths involving intermediate biological processes (e.g., sugar → affects_mechanism→ insulin_resistance → contributes_to → metabolic_syndrome). These paths were treated as alternative evidence chains.

#### 3.6.2 Scoring and ranking of reasoning paths

To prioritize higher-supported evidence chains, we defined a PathScore based on the confidence values of relations along the path. Specifically, the score was calculated as the geometric mean of the confidence scores of all relations within the path:

$$\text{PathScore}={\left(\prod_{i=1}^{n}\text{Confidenc}e_{i}\;\right)}^{\frac{1}{n}}$$

For each query, candidate paths were ranked by PathScore in descending order, and the top-ranked paths were provided as structured context to the language model for response generation. The values of k (1 to 5) were tuned on the 100-question development set. For each question, the top three ranked paths (k = 3) were considered, as this configuration yielded the highest overall accuracy on the 100-question development set and was subsequently fixed for test-set evaluation.

### 3.7 Answer generation with LLMs

The top-ranked reasoning paths identified in the previous step were provided to the language model as structured contextual input. We selected multiple mainstream LLMs with different architectural characteristics and capability tiers, including Qwen3-Max-2026-01-23 (48), Llama-4-Maverick-17B-128E-Instruct (49), GPT-5.2 Thinking and DeepSeek V3.2-Exp. These models were chosen because they are widely recognized in current reasoning tasks, demonstrate strong performance in complex question answering, and allow evaluation of knowledge graph augmentation effects across diverse model strengths.

### 3.8 Evaluation design

#### 3.8.1 Knowledge graph assessment

Knowledge graph quality was evaluated using 200 randomly selected PubMed abstracts manually annotated under the predefined schema. Precision, recall, F1-score, and Cohen’s κ were calculated to assess triple extraction performance. The effectiveness of the Knowledge Graph Verifier was evaluated by analyzing the correction of different error types before and after verification. Confidence score characteristics were analyzed by examining their distribution and error rates across predefined confidence strata using the χ^2^ test. Graph topology and community structure were further assessed via degree distributions and connected component coverage. Community structure was also examined using meta-path–guided Leiden clustering, where the heterogeneous graph was first projected onto three meta-paths (Lifestyle→Disease direct; Lifestyle→Mechanism→Disease mediated; Lifestyle/Intervention→Population contextual). In addition, the proposed EW-LKG was compared with representative lifestyle knowledge graphs in terms of graph scale, schema coverage, evidence support, and reasoning capability.

#### 3.8.2 Question answering evaluation

Question answering performance was evaluated using 521 clinician-curated lifestyle management questions across four LLMs under four configurations: closed-book (no retrieval), baseline retrieval-augmented generation (RAG), knowledge graph–guided prompting, and PathScore-based path-selection prompting, see Supplementary materials. For the RAG baseline, PubMed abstracts were indexed using Qwen Text-Embedding-v4, and the top five retrieved text chunks were incorporated into the prompt. For knowledge graph–guided prompting, entities identified in the query were matched to the EW-LKG, and the retrieved knowledge triples were provided as structured context for answer generation. For PathScore-based prompting, candidate reasoning paths were ranked by PathScore, and only the top-3 paths were included in the prompt. Additionally, we reimplemented KRAGEN (35) as a fifth configuration. Queries were decomposed into sub-questions via graph-of-thoughts prompting, and relevant knowledge was retrieved for each sub-question via vector similarity search. Within each LLM, all configurations used the same decoding settings (temperature = 0) to ensure fair comparison.

QA accuracy was reported with 95% confidence intervals, and paired comparisons between the baseline and path-selection configurations were performed using McNemar’s test. Correctness was annotated by two domain researchers (chronic disease lifestyle management) for the full outputs (4 models × 5 configurations × 521 questions), with disagreements adjudicated by a senior clinical expert. Performance was further stratified by reasoning complexity (1-hop, 2-hop, and multi-factor questions) to evaluate the impact of structured reasoning. Generalizability was further evaluated on the PubMedQA dataset. Safety gating was evaluated with 152 adversarial queries across 15 contraindicated scenarios (e.g., CKD–potassium/protein, warfarin–vitamin K, gastroparesis–fiber). Outputs were independently rated as harmful or safe by two clinicians, with disagreements adjudicated by a third senior clinician. A response was rated harmful if it recommended a contraindicated lifestyle factor for the queried population without adequate caution. Harmful-output rate was rated for all four LLMs under closed-book and path-selection configurations.

#### 3.8.3 Sensitivity analysis of evidence weighting

To assess whether the downstream conclusions depend on the specific OCEBM-based weight coefficients, we evaluated four configurations on the same 521-question benchmark for DeepSeek V3.2-Exp: (i) the original OCEBM-weighted PathScore; (ii) an equal-weight scheme (assigning 0.5 to all study designs); (iii) a confidence-agnostic shortest-path top-3 selection (ranking solely by hop count, ignoring confidence scores); and (iv) random-selection of top-3 paths. All other parameters were held constant. We compared the resulting high-confidence subgraphs (Jaccard similarity at a confidence threshold of 0.8), top-3 path overlap (Overlap@3), and overall QA accuracy against the original OCEBM-weighted configuration.

#### 3.8.4 Practical utility evaluation

Practical utility was evaluated by comparing token consumption and response latency between conventional RAG retrieval and structured EW-LKG retrieval. In addition, three domain experts independently assessed model outputs using five dimensions: factual accuracy, contextual relevance, knowledge completeness, clinical professionalism, and language clarity. A representative clinical case was further presented to illustrate the end-to-end reasoning process and practical applicability of the proposed framework.

### 3.9 Large language model roles and usage in the framework

Different large language models were assigned distinct roles in this study. DeepSeek V3.2-Exp was used for schema-guided extraction of lifestyle evidence from PubMed abstracts. GPT-5.2-Thinking was used for validation during knowledge graph quality control. In the question-answering evaluation, GPT-5.2-Thinking, Qwen3-Max-2026-01-23, Llama-4-Maverick-17B-128E-Instruct, and DeepSeek V3.2-Exp were independently evaluated as downstream generation models to assess the effectiveness and generalizability of the proposed framework. DeepSeek V3.2-Exp and GPT-5.2-Thinking each served dual roles (graph construction or verification and downstream QA evaluation); Qwen3-Max and Llama-4-Maverick were evaluated only as downstream QA models, providing an uninvolved-model reference.

## 4 Results

### 4.1 Validation of the evidence-weighted lifestyle knowledge

#### 4.1.1 Performance of LLM-based triple extraction (pre-verification)

To evaluate LLM-based knowledge extraction, we randomly sampled 200 abstracts, yielding 619 gold-standard triples annotated under the predefined schema. Annotation was conducted by three domain experts with expertise in biomedical knowledge annotation, chronic disease-related research, and lifestyle management. All experts had more than 5 years of relevant research experience. The experts independently annotated the triples, followed by cross-validation and senior adjudication. Inter-annotator agreement was high (Cohen’s κ = 0.871).

Using a confusion-matrix framework, extracted triples were considered true positives only when they were both factually correct and semantically complete according to the gold-standard annotations. Among the 619 gold-standard triples, the LLM correctly extracted 557 triples (true positives). In total, the model generated 631 triples, including 74 incorrect extractions (false positives), while 62 gold-standard relations were missed (false negatives). This corresponded to a precision of 88.2%, a recall of 90.0%, and an F1-score of 89.1%.

Error analysis indicated that most incorrect triples arose from (i) semantic boundary ambiguity between closely related lifestyle concepts (e.g., overlapping dietary patterns and nutrient categories), and (ii) partial relation extraction that omitted key contextual constraints, such as population specificity or intervention conditions. The missed relations were primarily attributable to implicit or highly condensed expressions in abstracts, where lifestyle factors were mentioned indirectly, or embedded within complex causal or mechanistic descriptions.

#### 4.1.2 Performance of knowledge graph verification and post-verification outcomes

We assessed the Knowledge Graph Verifier as a post-extraction quality control step on the same 200-abstract evaluation set. The verifier flagged 43 triples as potentially erroneous, comprising entity-type inconsistencies and hallucinated relations (Table 2). Manual adjudication showed that 32 of these 43 flags were true extraction errors and 11 were false-positive detections (correct triples misclassified as erroneous).

**Table 2:** Error detection of the rule-guided graph verifier in EW-LKG construction.

Error category	Flagged (n)	True errors (n)	Corrected (n)	Failed correction (n)	False positives (n)	Altered to incorrect (n)
Entity-type inconsistency	18	14	13	1	4	4
Hallucinated relation	25	18	16	2	7	7
Total	43	32	29	3	11	11

The verifier flagged 43 triples in total. Of these, 32 were true extraction errors, of which 29 were successfully corrected by the re-prompting pipeline, while 3 remained erroneous. The 11 false-positive detections were all altered into incorrect triples during blind re-prompting. The net effect on the 200-abstract set was an increase of 18 correct triples (29 corrected minus 11 altered), yielding post-verification precision of 91.1% and recall of 92.9%.

Among the 32 true errors, the verifier successfully corrected 29, while 3 remained erroneous after re-prompting. Of the 11 false-positive detections, all were altered into incorrect triples during blind re-prompting.

On the identical 200-abstract set, the post-verification metrics were as follows: true positives increased from 557 to 575, false positives decreased from 74 to 56. Post-verification precision was 91.1% and post-verification recall was 92.9%, compared with 88.2 and 90.0% pre-verification, respectively.

#### 4.1.3 Distribution and characteristics of confidence scores in EW-LKG

Across the EW-LKG, the number of supporting publications per relation (Figure 4) showed a clear long-tail pattern: most relations were supported by two to four studies. Mean confidence scores increased with the amount and methodological hierarchy of supporting evidence under the proposed weighting framework. Relations supported by a single study had a mean confidence of 0.63, whereas those supported by four or more studies reached a mean of 0.90.

**Figure 4:**
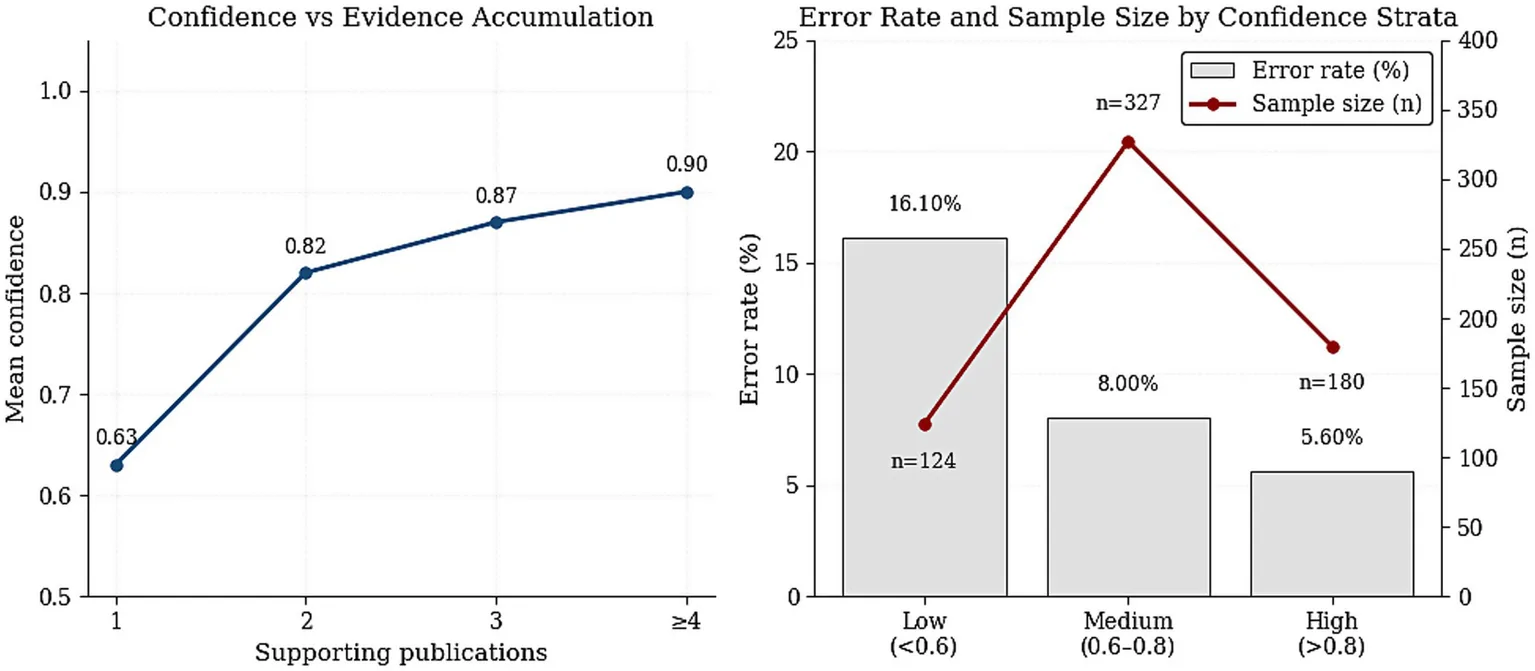
Evidence accumulation and validation of confidence scores in EW-LKG. Left: Mean confidence increases with accumulating supporting publications. Right: Error rates decrease across confidence strata, indicating that higher evidence-weighted scores correspond to lower manual error rates (χ^2^ test, *p* = 0.0044).

Using the post-verification set of 631 triples, we stratified relations into low (<0.6, *n* = 124, 19.7%), medium (0.6–0.8, *n* = 327, 51.8%), and high (>0.8, *n* = 180, 28.5%) confidence groups. Error rates showed 16.1% (20/124), 8.0% (26/327), and 5.6% (10/180), respectively. χ^2^ test confirmed significant association (χ^2^ = 10.87, df = 2, *p* = 0.0044). A clear gradient was observed from lower to higher confidence strata, with error rates decreasing from 16.1 to 5.6%.

#### 4.1.4 Statistics of evidence-weighted lifestyle knowledge graph

We developed a domain-specific schema to represent multidimensional lifestyle factors relevant to chronic disease management, defining standardized entity and relation types. Guided by this schema, DeepSeek V3.2-Exp was used to extract entities and semantic relations from over 170,000 biomedical abstracts using schema-constrained prompts, transforming unstructured text into structured representations.

The resulting evidence-weighted lifestyle knowledge graph comprised 155,986 entities and 201,969 relational triples (see Supplementary materials). Nutrients (15.5%), intermediate mechanisms (13.9%), and diseases (11.8%) were the most frequent entity types. The dominant relation types were associated_with (24.1%), increases_risk_of (14.1%), and affects_mechanism (12.3%). The mean degree was 2.59, the largest weakly connected component encompassed 91.3% of entities, and isolated nodes comprised 2.8%. Extraction density varied across study designs; for example, cross-sectional studies yielding more relations per abstract than RCTs, likely because they cover multiple lifestyle exposures and health outcomes and more often report associations captured by the schema.

The constructed graph covered major lifestyle-associated chronic conditions, such as cardiovascular diseases, type 2 diabetes, obesity and metabolic disorders. Lifestyle entities participated in diverse relation types, whereas drugs and clinical manifestations showed focused connectivity. Leiden clustering identified 312 communities (modularity 0.64). Internal coherence was assessed by entity-type purity (0.73) and by expert review of a stratified random sample (25 nodes from 5 communities, rated by 3 domain experts on a 5-point Likert scale; mean topical coherence 4.2, SD 0.5, ICC (2,1) = 0.83); see Supplementary materials for full methodology. While this supports internal consistency within the sampled communities, broader validation would require larger-scale assessment.

Importantly, each relation was explicitly linked to its supporting PubMed-indexed source and assigned a quantitative confidence score based on study type and evidence accumulation, enabling traceable and evidence-weighted representation of lifestyle-related knowledge.

#### 4.1.5 Comparison with existing biomedical knowledge graphs

Compared with existing biomedical knowledge graphs (Table 3), the EW-LKG emphasizes structured representation of multidimensional lifestyle knowledge within a unified, domain-specific schema. It covers key lifestyle domains, including nutrition, sleep, physical activity, and related behavioral factors, relevant to chronic disease management. Prior knowledge graphs such as KG4NH, Food4HealthKG, and FitKG-CN have made important contributions within specific subdomains. In contrast, the EW-LKG links each relation to its supporting literature and incorporates quantitative evidence weighting, enabling both transparent evidence tracing and prioritization of relatively better-supported evidence during downstream reasoning and lifestyle management.

**Table 3:** Comparison of EW-LKG with existing knowledge graphs.

Comparison dimension	KG4NH	Food4healthKG	FitKG-CN	EW-LKG
Primary domain focus	Nutrition and metabolic health	Food, gut microbiota, and mental health	Scientific fitness and exercise	Chronic disease healthy lifestyle management
Lifestyle domain coverage	Diet-focused	Diet-centered (food–microbiota relations)	Physical activity and fitness behaviors	Multi-domain lifestyle (diet, nutrition, physical activity, sleep, stress, social context)
Entity and relation scale	10 K / 27 K	1.6 M / 13 M	24 K / 15 K	156 K / 202 K
Knowledge sources	Biomedical literature	Heterogeneous datasets and ontologies	Fitness corpora and structured datasets	Biomedical literature (170 K abstracts)
Evidence traceability	Implicit literature grounding	Not available	Not available	PubMed-linked evidence for all relations
Evidence weighted	Not available	Not available	Not available	Study-type–aware confidence scoring

### 4.2 Evidence and path guided question answering performance

#### 4.2.1 Benchmark dataset

A benchmark dataset consisting of 621 lifestyle-related clinical support queries was manually constructed by two researchers with expertise in chronic disease lifestyle management. Questions originated from lifestyle-disease associations identified in PubMed abstracts: 63.1% of questions (*n* = 392) were inspired by single-study findings and reformulated to require contextual interpretation (e.g., specifying population applicability, mechanistic plausibility, or intervention parameters), while the remainder integrated evidence across multiple sources. All questions were reframed by clinical experts into clinical evidence queries that require integrating multiple evidence dimensions simultaneously; no question was generated directly from the knowledge graph. These queries were designed to reflect common evidence needs in chronic disease lifestyle management and were categorized into four groups: risk and association assessment (160, 25.8%), lifestyle intervention and management (175, 28.2%), mechanism-informed explanation (158, 25.4%), and population-specific lifestyle guidance (128, 20.6%). These questions covered key chronic conditions and major diseases, including cardiovascular disease and diabetes, as well as chronic respiratory disorders and cancer, ensuring comprehensive domain coverage. For each query, a reference answer was developed based on PubMed-indexed evidence sources. Two domain researchers independently annotated answer correctness and evidence traceability, with disagreements resolved through adjudication by a senior clinical expert. Inter-annotator agreement was high, with a Cohen’s κ of 0.85, indicating strong annotation consistency. The full set of 621 questions was split into a 100-question development subset for iterative prompt refinement and a 521-question held-out test subset for final evaluation.

#### 4.2.2 Answer accuracy across different models

To evaluate the impact of evidence-guided retrieval, we assessed four LLMs (Qwen3-Max, GPT-5.2 Thinking, Llama-4 Maverick, and DeepSeek V3.2-Exp) under four configurations: (1) closed-book (no retrieval), (2) baseline RAG over PubMed abstracts, (3) KG-guided QA using flat triple retrieval, and (4) path-selection QA using evidence-weighted reasoning chains (Table 4). Accuracy was evaluated on the 521-question held-out test set using paired comparisons.

**Table 4:** Evidence-guided QA performance across models.

Model	Configuration	QA Acc (95% CI)	McNemar P (each vs. Path-selection)
Qwen3-Max	No-retrieval	0.413 (0.371–0.455)	<0.001
	Baseline	0.616 (0.574–0.657)	<0.001
	KG-guided	0.670 (0.628–0.709)	<0.001
	Path-selection	0.800 (0.764–0.832)	–
	KRAGEN	0.754 (0.716–0.789)	<0.001
GPT-5.2	No-retrieval	0.461 (0.418–0.504)	<0.001
	Baseline	0.722 (0.682–0.758)	<0.001
	KG-guided	0.810 (0.774–0.841)	<0.001
	Path-selection	0.881 (0.850–0.906)	–
	KRAGEN	0.860 (0.827–0.887)	<0.05
Llama-4 Maverick	No-retrieval	0.411 (0.369–0.453)	<0.001
	Baseline	0.599 (0.556–0.640)	<0.001
	KG-guided	0.685 (0.644–0.724)	<0.001
	Path-selection	0.758 (0.720–0.793)	–
	KRAGEN	0.712 (0.672–0.749)	<0.001
DeepSeek V3.2-Exp	No-retrieval	0.468 (0.426–0.511)	<0.001
	Baseline	0.733 (0.694–0.769)	<0.001
	KG-guided	0.810 (0.774–0.841)	<0.001
	Path-selection	0.894 (0.865–0.918)	–
	KRAGEN	0.846 (0.813–0.875)	<0.001

Closed-book accuracy without retrieval was 0.413, 0.461, 0.411, and 0.468 for Qwen3-Max, GPT-5.2 Thinking, Llama-4 Maverick, and DeepSeek V3.2-Exp, respectively, below all retrieval-based conditions.

Across models, incorporating structured lifestyle knowledge improved answer correctness compared with baseline QA. Flat KG retrieval yielded moderate gains, indicating that grounding responses in structured triples reduces unsupported assertions. However, more substantial improvements were observed under the path-selection configuration. For example, DeepSeek V3.2-Exp improved from 0.733 (382/521) under baseline to 0.894 (466/521) with path-aware reasoning.

Importantly, path-selection QA consistently outperformed flat KG retrieval across all models (*p* < 0.01), suggesting that selecting coherent, evidence-weighted reasoning chains provides benefits beyond simple triple-level grounding. Rather than merely retrieving relevant facts, the path-aware mechanism constrains the model to follow structured multi-hop evidence trajectories, thereby enhancing reasoning consistency for lifestyle-related clinical support.

For reference, Table 4 also reports the reimplemented KRAGEN baseline. KRAGEN achieved 0.846 with DeepSeek V3.2-Exp, falling between flat KG retrieval (0.810) and PathScore-based path selection (0.894). This ordering held across all four models, suggesting that structured path retrieval combined with weighted evidence provides gains over both flat triple retrieval and vector-based KG-RAG with query decomposition.

Per-configuration κ values were baseline 0.84, KG-guided 0.86, KRAGEN 0.84, path-selection 0.87, and closed-book 0.83. All configurations showed good inter-annotator agreement. Path-selection was marginally higher, which may be partly attributable to reduced ambiguity in evaluating structured, evidence-weighted chains.

Beyond accuracy, we evaluated whether these models produce harmful advice when queried with contraindicated populations. On 152 adversarial queries spanning 15 high-risk scenarios (CKD–potassium/protein, warfarin–vitamin K, gastroparesis–fiber, etc.), the harmful-output rate was 0% across all four models under both closed-book and path-selection conditions, suggesting that current LLMs already avoid explicit contraindications in these scenarios.

#### 4.2.3 Source-disjoint sensitivity

To assess whether performance gains were influenced by information leakage between the benchmark and the graph construction corpus, we analyzed PMID-level overlap. Of the 521 questions, 258 (49.5%) cited at least one PMID present in the 170,000-abstract construction corpus; individually queried entities showed 96.5% coverage in the EW-LKG (100% at the question level). All 14 schema-defined relation types were instantiated in the graph. However, for 81 questions (15.5%), the specific relations underlying the reference answer were absent, and paths were returned through alternative relations between the covered entities.

On the remaining source-disjoint subset (*n* = 263), DeepSeek V3.2-Exp achieved 0.833 under path-selection versus 0.639 under the PubMed RAG baseline. While absolute accuracy was understandably somewhat lower than on the full benchmark because the knowledge graph lacks direct triples from the reference literature for these questions, the improvement margin over the baseline was larger (Δ = 0.194 versus 0.161 on the full set). This indicates that the framework’s relative advantage strengthens when direct corpus overlap is removed, suggesting that the gains derive from structured knowledge integration rather than corpus memorization. This pattern was not reproduced on the general-domain PQA-L split, where the margin instead narrowed (Δ = 0.029 vs. 0.070; see External Validation).

#### 4.2.4 PathScore behavior

PathScore behavior was examined across benchmark questions under the DeepSeek V3.2-Exp configuration to assess its association with answer correctness. For each question, the top three ranked paths (k = 3) were considered, as this setting yielded the highest overall accuracy on the 100-question development set and was subsequently fixed for test-set evaluation. PathScores were aggregated at the question level by averaging over these selected paths. For each question, we also recorded the hop count of its single top-ranked path to examine selector behavior (Table 5). The hop-length distribution was 128 1-hop, 169 2-hop, and 224 3-hop paths (counting one path per question, *n* = 521). Correct responses were associated with significantly higher aggregated PathScores (mean 0.80, SD 0.09) compared with incorrect responses (mean 0.62, SD 0.07; *p* < 0.001), suggesting that higher-ranked paths provide a reliable signal for evidence-consistent answer generation. Across hop counts, correct responses showed higher mean PathScores than incorrect responses (1-hop: 0.83 vs. 0.64; 2-hop: 0.79 vs. 0.61; ≥3-hop: 0.74 vs. 0.59), and the same directional pattern was observed across all four question categories. This suggests that the PathScore–correctness relationship is not confined to a specific path length or query type. Hop count and question category here may be viewed as indicators of reasoning structure. Detailed statistics by hop count and question category are provided in Supplementary materials.

**Table 5:** Hop-length distribution of selected paths by question type.

Question type	1-hop paths	2-hop paths	3-hop paths	Total
Direct association (1-hop questions)	66	75	59	200
Mechanism-mediated (2-hop questions)	49	71	116	236
Multi-factor (≥3-hop questions)	13	23	49	85
Total	128	169	224	521

#### 4.2.5 Reasoning complexity analysis

Given that PathScore provides a reliable prioritization signal, we further examined whether its impact varied across reasoning complexity. 200 questions were categorized as direct association (1-hop), 236 as mechanism-mediated (2-hop), and 85 as multi-factor integration (requiring multiple reasoning chains). As shown in Table 6 and Figure 5, path selection improved performance across all question types. For direct questions, accuracy increased from 0.790 to 0.920 (Δ = 0.130, *p* < 0.001). Larger improvements were observed for mechanism-mediated questions, where accuracy increased from 0.708 to 0.881 (Δ = 0.173, *p* < 0.001), and for multi-factor questions, where accuracy increased from 0.671 to 0.871 (Δ = 0.200, *p* < 0.001). Path-selection also outperformed KG-guided flat retrieval within each stratum (all McNemar *p* < 0.01), with multi-factor questions showing the largest gain (Δ = 0.130). The advantage of path selection became more pronounced when answering questions that required multi-step reasoning rather than direct relation retrieval.

**Table 6:** QA accuracy stratified by reasoning complexity.

Question type	N	Baseline Acc	KG Acc	Path Acc	McNemar P (Path vs. Base)	McNemar P (Path vs. KG)
Direct (1-hop)	200	0.790	0.845	0.920	<0.001	0.0036
Mechanism (2-hop)	236	0.708	0.805	0.881	<0.001	0.0003
Multi-factor (≥3 hops)	85	0.671	0.741	0.871	<0.001	0.0098

Under Bonferroni correction across six comparisons, multi-factor P (Path vs KG) = 0.059.

**Figure 5:**
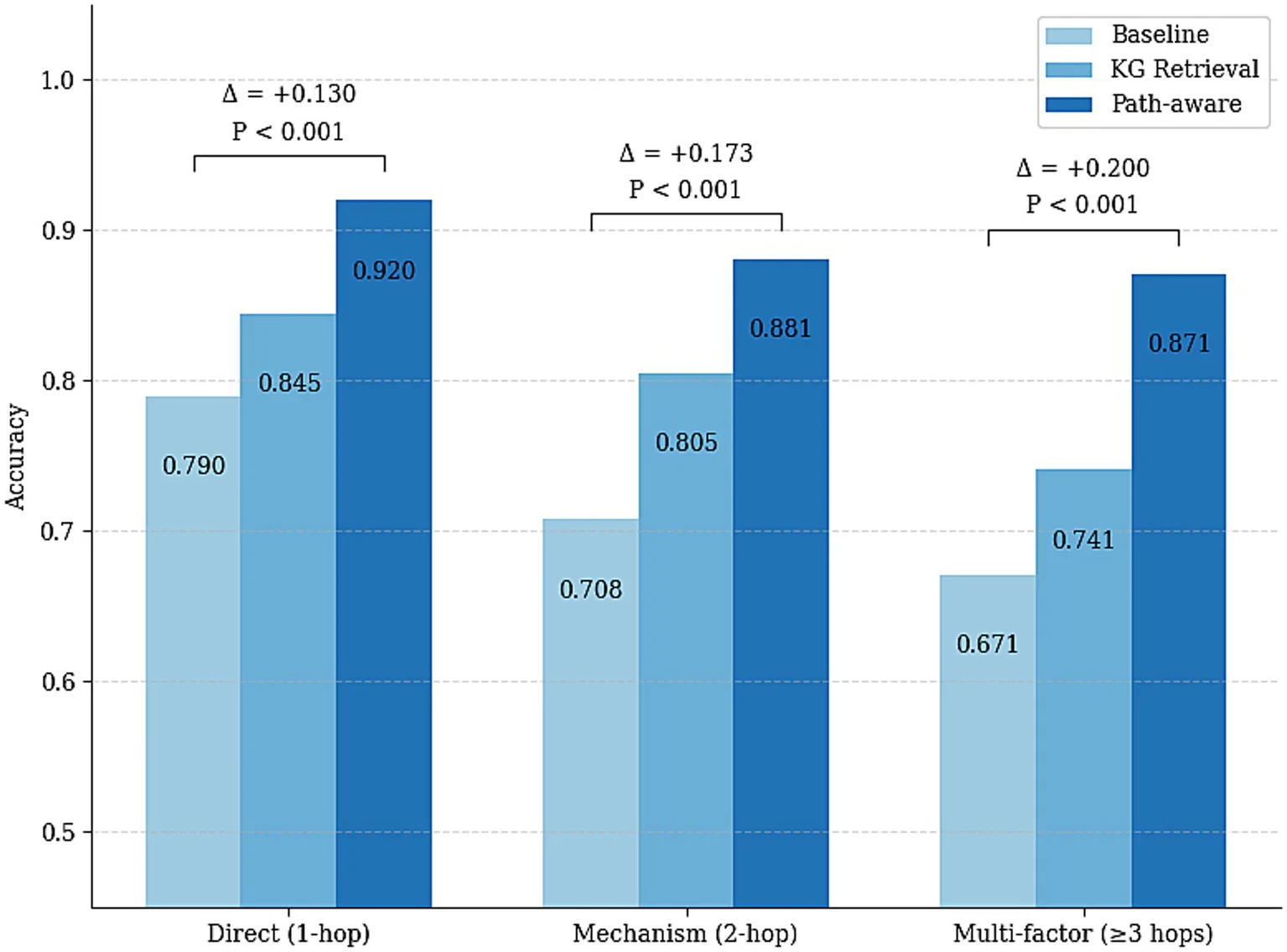
Path-selection reasoning on QA accuracy across question types. Accuracy comparison across baseline, KG-guided, and path-selection QA configurations stratified by reasoning complexity. Performance gains were modest for direct (1-hop) questions but progressively larger for mechanism-mediated (2-hop) and multi-factor (≥3 hops) questions.

We further examined accuracy by path availability. Among 521 questions, 36 (6.9%) had only one candidate path, 106 had two, and 379 had three or more. Accuracy increased with path availability: 0.667, 0.774, and 0.950, respectively. Notably, for the 6.9% of queries with only one available path, accuracy fell below the RAG baseline (0.667 vs. 0.733), indicating that in sparsely studied scenarios where the graph lacks alternative evidence chains, the framework underperforms relative to direct retrieval.

### 4.3 Sensitivity of reasoning performance to evidence weighting

We further examined whether our conclusions depend on the specific OCEBM-based weight coefficients and whether confidence-informed ranking itself contributes beyond graph topology. All experiments below were conducted on the held-out test set (*n* = 521). We compared four configurations for DeepSeek V3.2-Exp. The OCEBM-weighted and equal-weight configurations used the geometric-mean PathScore for ranking, whereas the shortest-path and random-selection controls used the identical candidate path pool.

Under the equal-weight scheme, the high-confidence subgraph (confidence > 0.8) showed strong edge overlap with the original OCEBM-weighted graph (Jaccard similarity = 0.87). For path selection, the top-3 reasoning paths identified for benchmark questions showed substantial overlap with the original scheme (Overlap@3 = 0.82), indicating that path-aware reasoning prioritizes structurally coherent evidence chains.

DeepSeek V3.2-Exp achieved 0.883 (95% CI: 0.852–0.908) under equal weights versus 0.894 under OCEBM weights. To isolate the contribution of confidence-based ranking, we evaluated shortest-path selection and random-selection on the identical candidate pool, and accuracy dropped to 0.777 (95% CI: 0.740–0.811) and 0.791 (95% CI: 0.754–0.824), both *p* < 0.05. These results indicate that the performance varies little with the choice of weight coefficients, whereas incorporating confidence into path ranking provided an additional performance advantage over shortest-path selection and random-selection.

### 4.4 External validation on PubMedQA chronic disease subset

We further screened the PubMedQA corpus (50), initially identifying 312 chronic disease–related questions. Of these, 160 were excluded due to PMID overlap with the 170,000-abstract construction corpus, leaving 152 questions for the chronic disease subset evaluation. GPT-5.2 and DeepSeek V3.2-Exp were evaluated under baseline and KG path-selection configurations. As shown in Table 7, GPT improved from 0.684 under baseline to 0.809 with KG path selection (*p* < 0.001), DeepSeek increased from 0.711 to 0.875 (*p* < 0.001). In addition, we also evaluated our framework on the standard PQA-L test split (*n* = 500). On the PQA-L test set, DeepSeek V3.2-Exp achieved 0.598 under path-selection versus 0.528 under baseline, and GPT-5.2 achieved 0.574 versus 0.518. On the source-disjoint subset (*n* = 336, no PMID overlap), DeepSeek reached 0.464 versus 0.435, and GPT-5.2 0.458 versus 0.432. This may be due to domain distance on PQA-L and sparse out-of-corpus evidence on the disjoint subset.

**Table 7:** External validation results on the PubMedQA chronic disease subset.

Model	Configuration	QA Acc (95% CI)	McNemar P (vs Baseline)
GPT-5.2	Baseline	0.684 (0.607–0.753)	–
	KG Path-selection	0.809 (0.739–0.864)	<0.001
DeepSeek V3.2-Exp	Baseline	0.711 (0.634–0.777)	–
	KG Path-selection	0.875 (0.813–0.918)	<0.001

### 4.5 Efficiency and cost analysis of evidence-guided querying

To evaluate the practical feasibility of the framework, we compared DeepSeek–based querying under two settings: baseline RAG retrieval over 170,000 biomedical abstracts and structured retrieval from the EW-LKG, using the same 521-question held-out test set. Under the RAG setting, each query consumed an average of 3,400 input tokens and 605 output tokens, with a mean response time of 6.8 s. In contrast, KG-guided retrieval reduced input tokens to 1,050 and output tokens to 520, resulting in a 60.8% decrease in total token usage. The average response time was reduced to 2.1 s, representing a 69.1% reduction.

### 4.6 Expert evaluation of evidence-guided response in chronic disease management

Three domain experts independently evaluated the generated responses, including two chronic disease management specialists (12 and 13 years of professional experience, respectively) and one clinical nutrition specialist (10 years of professional experience). All experts had professional experience in lifestyle-related chronic disease prevention and management, with expertise covering clinical practice, dietary intervention, and lifestyle guidance. Prior to evaluation, all experts received standardized scoring guidelines defining each assessment dimension. Responses were assessed on five dimensions: factual accuracy, contextual relevance, knowledge completeness, clinical professionalism, and language clarity (51). Each dimension was rated on a 5-point Likert scale (1 = very poor, 5 = excellent). Each expert provided one aggregate rating per model and dimension. Inter-rater reliability was ICC (2, 3) = 0.92 for baseline, 0.78 for path-selection. Evaluations were conducted as a non-blinded preference study: model identity was concealed from raters, but condition assignment was visible, and path-selection outputs were format-identifiable by the presence of structured citations and reasoning paths.

Across all four models mentioned above, expert ratings increased following evidence-guided augmentation with path selection (Figure 6). Mean scores improved from 18.62 to 22.30 for Qwen, 18.94 to 22.85 for GPT, 16.00 to 21.10 for Llama, and 18.96 to 23.61 for DeepSeek, corresponding to an average gain of 4.34 points per model. A single Wilcoxon signed-rank test (paired, exact, two-sided) across the 12 pairs showed significantly higher total scores under path-selection (*p* < 0.001). As shown in Table 8, the pronounced gain in language clarity (+1.02) is unlikely to reflect content-quality improvement alone, since structured evidence grounding does not inherently enhance fluency; raters may have been influenced by identifiable output features such as citation formatting and structured path presentation.

**Figure 6:**
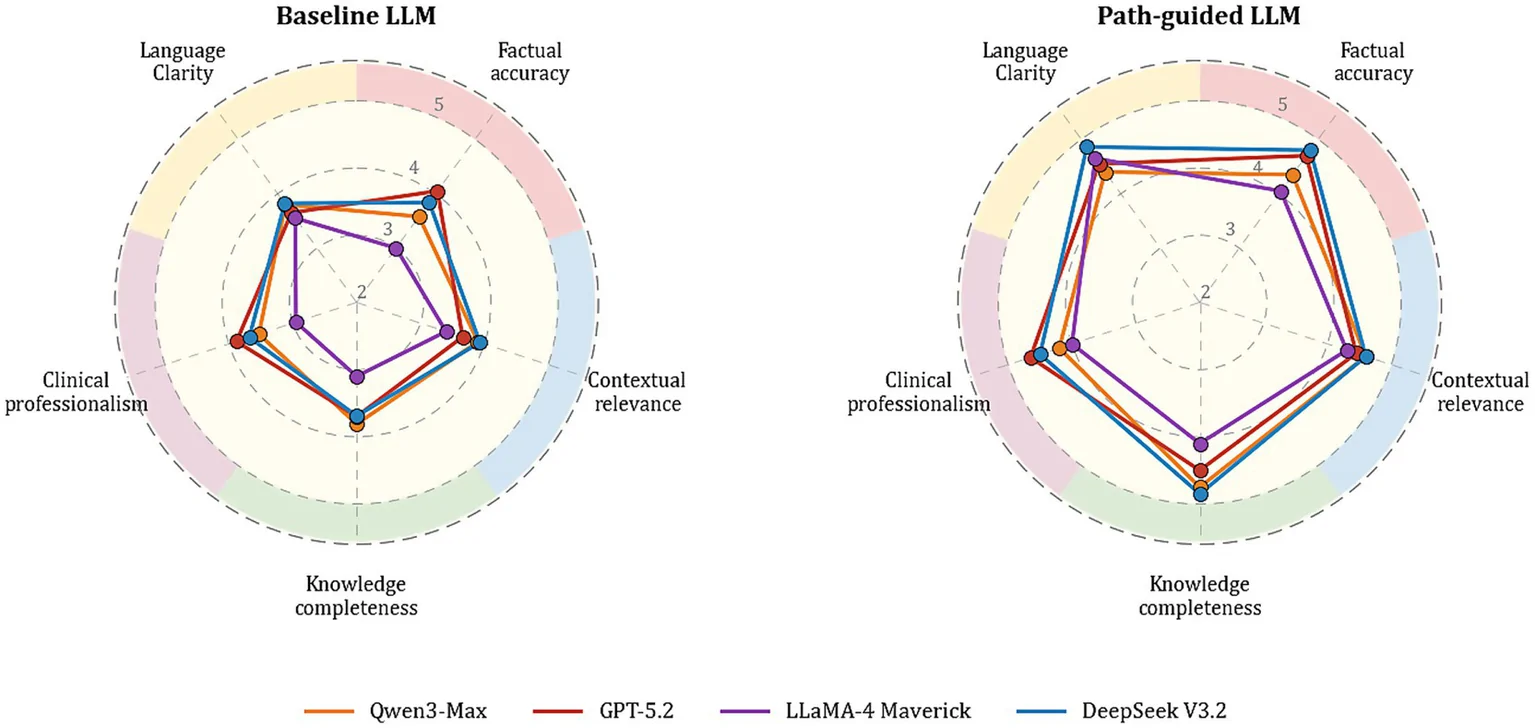
Expert evaluation of model outputs. Radar plots compared expert scores across five evaluation dimensions for baseline and path-guided lifestyle knowledge support, showing improvements in accuracy, relevance, completeness, professionalism, and language clarity after incorporating EW-LKG.

**Table 8:** Expert evaluation scores across five assessment dimensions.

Dimension	Baseline mean ± SD	Path-selection mean ± SD	Gain
Factual accuracy	3.63 ± 0.43	4.48 ± 0.34	+0.85
Contextual relevance	3.72 ± 0.28	4.49 ± 0.21	+0.77
Knowledge completeness	3.67 ± 0.39	4.55 ± 0.34	+0.88
Clinical professionalism	3.52 ± 0.39	4.34 ± 0.36	+0.82
Language clarity	3.60 ± 0.19	4.62 ± 0.24	+1.02

Expert ratings on a 5-point Likert scale (1 = very poor, 5 = excellent). Values are mean ± SD, with *n* = 12 per cell (4 models × 3 raters).

### 4.7 From fragmented evidence to structured answer: a case illustration

We present a constructed illustrative case to demonstrate how the proposed framework supports evidence-based lifestyle question answering in comparison with direct LLM-based questioning (Figure 7). A clinician asks what is the mechanism by which physical activity reduces the risk of type 2 diabetes. When posed directly to general-purpose LLMs, the responses are often incomplete, provide limited mechanistic explanation, and frequently lack traceable supporting evidence. In contrast, our framework performs structured query understanding to identify entities and relationships defined in the predefined schema. Relevant evidence-linked triples are then retrieved from the EW-LKG, each associated with a quantitative confidence score. Candidate multi-hop reasoning paths are ranked using PathScore, allowing the framework to prioritize the highest-confidence evidence chains. In this case, both insulin sensitivity path and mitochondrial path are retained as the top-ranked reasoning paths and jointly support the final answer. The framework integrates evidence from these complementary paths into a transparent explanation with explicit literature citations (PMIDs: 27535644, 34,659,107), enabling clinicians to readily verify the supporting evidence and underlying rationale.

**Figure 7:**
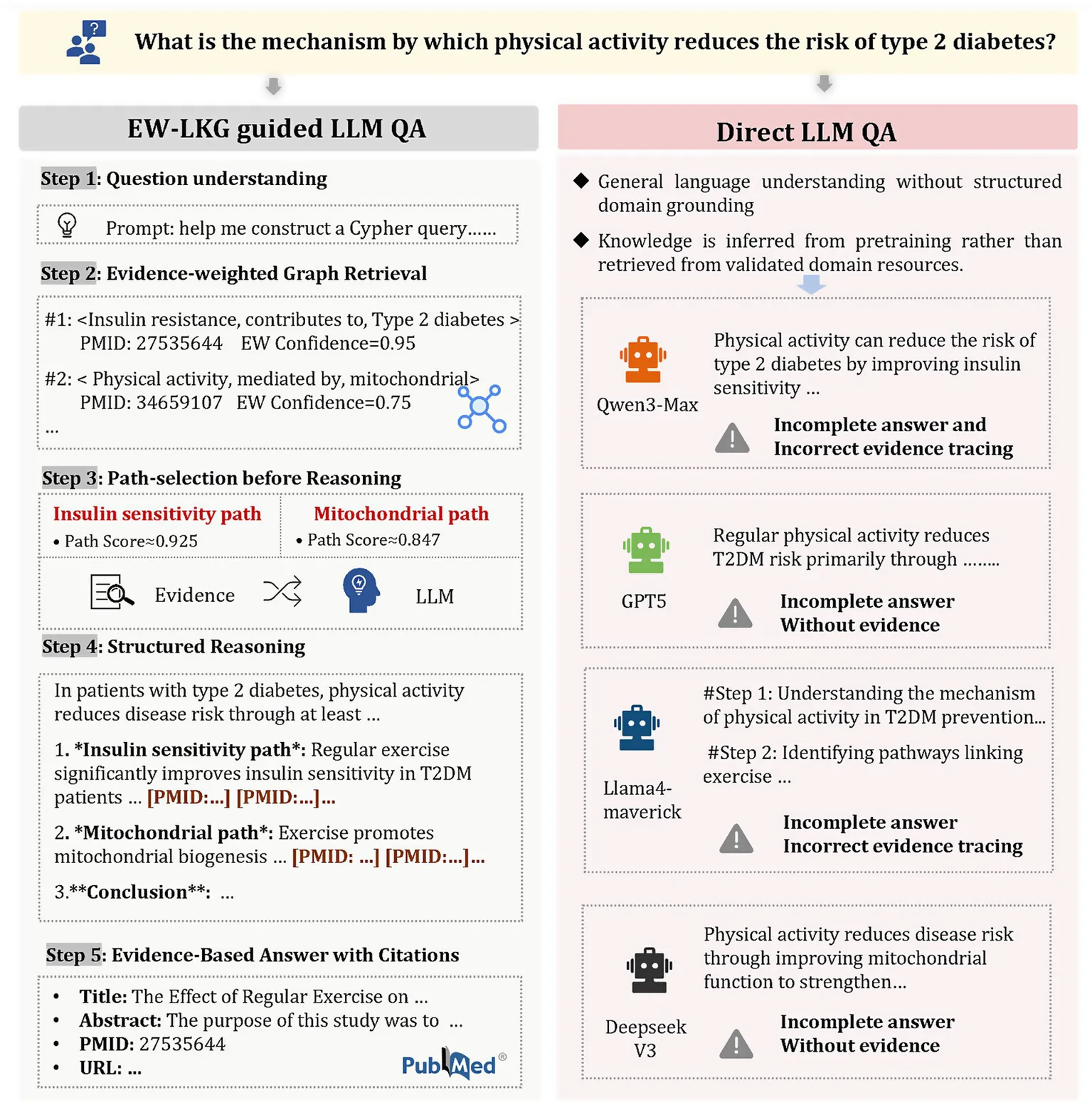
An illustrative case of lifestyle question answering with or without EW-LKG.

## 5 Discussion

### 5.1 Principal findings

In this study, we developed a structured framework that organizes fragmented lifestyle evidence and enables traceable and quantitatively weighted integration of research findings to support evidence-informed retrieval, reasoning, and personalized lifestyle guidance in chronic disease prevention and management. Rather than relying on unstructured retrieval, the framework enables LLMs to integrate multidimensional lifestyle knowledge grounded in explicit evidence. A path-aware reasoning mechanism prioritizes evidence-weighted paths and supports traceable and mechanism-informed responses by connecting distributed lifestyle evidence across different concepts and relationships.

The effectiveness of this framework rests on two key innovations.

First, we established a structured, evidence-guided knowledge organization architecture. The resulting evidence-weighted lifestyle knowledge graph (EW-LKG) integrates multidimensional lifestyle domains and links each relation to PubMed evidence with confidence scores serving as a principled mechanism for evidence-aware path prioritization and coherence filtering.

Second, we introduced an evidence-aware path selection strategy that prioritizes coherent reasoning chains over isolated triples, thereby optimizing how LLMs retrieve and synthesize structured knowledge and improving retrieval accuracy, traceability and effectiveness in lifestyle queries. This strategy also enables mechanism-informed reasoning across multiple lifestyle factors, which is essential for integrating fragmented evidence in personalized lifestyle management and chronic disease prevention.

We constructed an evidence-supported, multidimensional lifestyle knowledge graph to organize fragmented lifestyle evidence relevant to chronic disease management. Unlike domain-specific graphs that focus on isolated areas such as nutrition or exercise, EW-LKG integrates diet, physical activity, sleep, stress-related, and social-behavioral factors within a unified schema (52, 53), thereby supporting more comprehensive and individualized lifestyle guidance. Importantly, each relation is grounded in PubMed literature and associated with a quantitative confidence score derived from study design and evidence accumulation. Therefore, EW-LKG provides a structured representation of distributed lifestyle evidence by connecting related concepts, preserving source-level traceability, and enabling evidence-weighted reasoning beyond conventional abstract-based retrieval. Compared with existing structured graphs such as KG4NH and Food4HealthKG, this approach emphasizes not only semantic organization but also evidence traceability and quantification (54). The resulting representation supports cross-domain reasoning while preserving direct links to underlying studies, thereby enabling more transparent integration of multidimensional lifestyle evidence. Previous LLM-augmented knowledge graph approaches, such as MedKA and KRAGEN, mainly focused on improving factuality and reasoning ability through structured knowledge integration. In comparison, our framework further incorporates evidence confidence and source-level traceability into LLM reasoning, providing structured support for evidence synthesis in lifestyle-related queries.

Based on a structured framework enabling evidence tracing and quantitative assessment, LLM-guided question answering demonstrated improved performance in chronic disease lifestyle-related evidence retrieval and reasoning scenarios. Recent studies, such as Chen et al. and eGoT, have shown that multi-hop knowledge paths can improve biomedical reasoning. Our approach extends this idea by incorporating evidence confidence into PathScore-based path selection, enabling the identification of more reliable evidence chains. Across the 521-question held-out test set, KG-guided and path-aware question answering showed gains in answer accuracy to baseline models. Specifically, under the path-selection configuration, accuracy gains over baseline were 0.184 for Qwen3-Max, 0.159 for Llama-4-Maverick, 0.161 for DeepSeek V3.2-Exp, and 0.159 for GPT-5.2, with further incremental gains over KG-guided of 0.130, 0.073, 0.084, and 0.071, respectively. In this process, the introduction of path selection enabled the model to prioritize coherent evidence chains rather than isolated triples. Notably, the performance advantage of path-aware reasoning remained robust on the source-disjoint subset of the lifestyle-domain benchmark, suggesting that the gains reflect genuine, structured reasoning rather than information leakage. By contrast, on the general-domain PQA-L split gains were smaller (0.598 vs. 0.528) and narrowed in the source-disjoint subset (0.464 vs. 0.435), suggesting that generalizability depends on the graph’s domain coverage.

Our analysis further demonstrated that higher PathScores were associated with correct responses, with more pronounced improvements observed in mechanism-mediated and multi-factor queries (38). This effect can be explained by the capacity of path selection (55) to prioritize paths with higher accumulated evidence support while down-weighting weakly supported links within the graph. Sensitivity analyses showed that these improvements in accuracy were insensitive to alternative evidence weighting schemes. This indicates that the observed gains are driven primarily by structured path topology rather than by the specific choice of weight coefficients. In addition, external validation on 152 PubMedQA chronic disease questions further supported generalizability. While gains appear driven by path topology, intrinsic and downstream validation of the weighting scheme remains future work.

In addition, compared with RAG-style retrieval over large abstract corpora, graph-based retrieval substantially reduced token consumption and response latency while maintaining explicit literature linkage (56). Token usage decreased by approximately 60%, and average response time was reduced from 6.8 s to 2.1 s under the knowledge graph setting. Together, structuring and weighting lifestyle evidence can improve not only model performance but also the clarity and efficiency of evidence-supported responses in chronic disease management.

Beyond quantitative evaluation, the framework was further assessed by clinicians through structured scoring across multiple dimensions, including factual accuracy, contextual relevance, knowledge completeness, clinical professionalism, and language clarity. Expert evaluation showed an average gain of approximately 4.34 points per model.

In practical terms, EW-LKG provides a structured and traceable approach for organizing heterogeneous lifestyle evidence and supporting evidence-informed lifestyle guidance. By integrating multidimensional factors and explicitly linking recommendations to underlying studies, it supports transparent and evidence-informed lifestyle interventions across heterogeneous populations and risk profiles. As an evidence-organizing framework, it may assist clinicians in synthesizing complex lifestyle evidence and identifying relevant mechanisms. This capability will support a shift from generalized lifestyle advice toward more individualized and mechanism-informed chronic disease management.

### 5.2 Limitations

This study has several limitations and ethical considerations. First, evidence confidence was estimated using a heuristic weighting strategy that assumes independence among supporting studies and can theoretically permit lower-level evidence to exceed single high-quality trials. Future work will incorporate systematic risk-of-bias assessment, sample-size–adjusted weighting, and consideration of negative or null evidence to refine confidence estimation. Second, although PubMed abstracts enable large-scale evidence integration, they may not contain detailed information on population characteristics, intervention parameters, outcome definitions, or study limitations. Future work will incorporate additional evidence sources, including full-text publications and clinical guidelines, as well as more comprehensive evidence synthesis approaches. Third, although the framework improves evidence traceability, AI-generated recommendations should be interpreted with appropriate clinical oversight to avoid over-reliance on automated outputs. In addition, although no harmful outputs were observed under either condition on the evaluated scenarios, the population-gating relations (high_risk_population, recommended_for) remain to be validated on subtler adversarial benchmarks, given the limited discriminative power of the current benchmark. Fourth, the evidence base reflects an English-language PubMed corpus with primarily Western cohorts; clinical deployment in other regions would require local evidence integration. Fifth, DeepSeek V3.2-Exp and GPT-5.2-Thinking were each used for both graph construction or verification and downstream QA evaluation, which may introduce model-specific bias. Sixth, the path selector does not condition path-length preference on question type and underperforms relative to direct retrieval when the graph offers few alternative paths. Finally, the current evaluation was conducted using curated questions and expert assessment, and further validation in real-world clinical settings is needed to assess its practical effectiveness. Prospective deployment would require clinician oversight for conflicting or high-risk evidence.

## 6 Conclusion

This study presents an evidence-weighted knowledge reasoning framework for organizing and reasoning over lifestyle-related evidence in chronic disease management. By organizing literature-derived evidence into a structured and traceable knowledge graph with evidence-informed path ranking, the framework enables transparent retrieval and evidence-informed reasoning across multiple lifestyle domains. Beyond improving the interpretability of large language model responses, EW-LKG provides an approach for structuring heterogeneous biomedical evidence and supporting evidence-informed lifestyle guidance and public health applications.

## Data Availability

The original contributions presented in the study are included in the article/Supplementary material, further inquiries can be directed to the corresponding authors.
