# Supplementary material for: An evidence-weighted knowledge framework with path-aware reasoning for personalized lifestyle management in chronic disease
Source: Front Public Health. 2026 Sep 24;14:1924985. doi: 10.3389/fpubh.2026.1924985 (PMC13646055; doi:10.3389/fpubh.2026.1924985)
Supplement: Supplementary file 1 [file Supplementary_file_1.DOCX]

Supplementary Material

# 1  The schema of Lifestyle-KG.

Lifestyle-KG schema in our study defined 13 entity classes capturing essential health and lifestyle elements. It also specified 14 relationship types describing interactions between entities. By formally defining these connections, this schema can support structured reasoning, evidence-based question answering, and multi-dimensional analysis.

Table S1. Schema of 13 entity classes in Lifestyle-KG

| **Type** | **Name** | **Definition** | **Example** |
| --- | --- | --- | --- |
| Entity | Disease | A chronic or acute pathological condition characterized by abnormal physiological processes and associated health risks. | “Type 2 diabetes mellitus is a major risk factor for cardiovascular complications.” |
| Entity | Health Outcome | A measurable health-related endpoint reflecting disease risk, progression, prognosis, or prevention outcomes. | “Improved glycemic control was associated with reduced cardiovascular risk.” |
| Entity | Intermediate Mechanism | A biological or physiological process that mediates the relationship between lifestyle factors and disease outcomes. | “Insulin resistance plays a central role in the development of type 2 diabetes.” |
| Entity | Population | A defined group characterized by demographic, clinical, or risk-related attributes relevant to health management. | “Older adults with obesity are at increased risk of metabolic disorders.” |
| Entity | Dietary Pattern | A habitual combination of foods and dietary behaviors reflecting overall eating habits. | “Adherence to a Mediterranean diet was associated with lower incidence of cardiovascular disease.” |
| Entity | Nutrient | A specific dietary component required for normal physiological function or implicated in disease risk. | “High sodium intake was associated with elevated blood pressure.” |
| Entity | Physical Activity | Any structured or unstructured bodily movement that contributes to energy expenditure and influences health outcomes. | “Regular moderate-to-vigorous physical activity reduced the risk of type 2 diabetes.” |
| Entity | Sleep Behavior | Characteristics of sleep patterns, including duration, quality, and timing, relevant to health and disease. | “Short sleep duration was associated with increased risk of metabolic syndrome.” |
| Entity | Stress & Mental State | Psychological or emotional conditions that influence physiological regulation and disease susceptibility. | “Chronic psychological stress was linked to increased inflammatory markers.” |
| Entity | Social & Behavioral Context | Social, environmental, or behavioral conditions that shape lifestyle behaviors and health outcomes. | “Social isolation was associated with poorer adherence to healthy lifestyle behaviors.” |
| Entity | Clinical Manifestation | Observable signs or symptoms reflecting disease presence, progression, or complications. | “Patients commonly presented with fatigue and polyuria.” |
| Entity | Intervention | A non-pharmacological or behavioral strategy aimed at modifying lifestyle factors or disease risk. | “Lifestyle intervention focusing on diet and physical activity improved metabolic outcomes.” |
| Entity | Drug | A pharmacological agent used for disease treatment or risk management, often considered in conjunction with lifestyle factors. | “Metformin therapy significantly improved glycemic control.” |

**Table S2.** Schema of 14 relationships in Lifestyle-KG

| **Type** | **Optimized Name** | **Definition** | **Domain** | **Range** | **Example** |
| --- | --- | --- | --- | --- | --- |
| Relationship | associated_with | Indicates a reported association between two entities without explicit causal direction. | Any Lifestyle Factor | Disease / Health Outcome | “Short sleep duration was associated with an increased risk of type 2 diabetes.” |
| Relationship | increases_risk_of | Indicates that an entity is reported to elevate the risk of a disease or adverse outcome. | Lifestyle Factor / Nutrient | Disease / Health Outcome | “High sodium intake increases the risk of hypertension.” |
| Relationship | reduces_risk_of | Indicates that an entity is reported to reduce disease risk or improve outcomes. | Lifestyle Factor / Intervention | Disease / Health Outcome | “Regular physical activity reduces the risk of cardiovascular disease.” |
| Relationship | affects_mechanism | Indicates that an entity influences a biological or physiological mechanism. | Lifestyle Factor / Nutrient | Intermediate Mechanism | “Chronic stress affects inflammatory pathways.” |
| Relationship | mediated_by | Indicates that an effect is explained through an intermediate biological mechanism. | Lifestyle Factor | Intermediate Mechanism | “The effect of sleep deprivation on diabetes risk is mediated by insulin resistance.” |
| Relationship | contributes_to | Indicates that a mechanism or condition contributes to disease development or progression. | Intermediate Mechanism | Disease | “Insulin resistance contributes to the development of type 2 diabetes.” |
| Relationship | leads_to | Indicates progression from disease to a clinical manifestation or outcome. | Disease | Clinical Manifestation / Health Outcome | “Type 2 diabetes leads to microvascular complications.” |
| Relationship | has_clinical_manifestation | Links a disease to its observed signs or symptoms. | Disease | Clinical Manifestation | “Hypertension is characterized by persistently elevated blood pressure.” |
| Relationship | recommended_for | Indicates that an intervention or lifestyle factor is recommended for a specific population. | Intervention / Lifestyle Factor | Population | “Lifestyle modification is recommended for adults at high cardiometabolic risk.” |
| Relationship | high_risk_population | Identifies populations at elevated risk for a disease or outcome. | Disease | Population | “Older adults with obesity represent a high-risk population for metabolic syndrome.” |
| Relationship | targets | Indicates that an intervention or drug targets a disease or mechanism. | Drug / Intervention | Disease / Intermediate Mechanism | “Metformin targets insulin resistance in patients with type 2 diabetes.” |
| Relationship | improves_outcome_of | Indicates improvement in a disease-related health outcome. | Intervention / Lifestyle Factor | Health Outcome | “Dietary intervention improved glycemic control.” |
| Relationship | worsens_outcome_of | Indicates deterioration of a disease-related outcome. | Lifestyle Factor | Health Outcome | “Sleep deprivation worsened metabolic outcomes.” |
| Relationship | supported_by | Links a relationship or claim to its supporting literature source. | Any Entity or Relation | Evidence Source (Abstract) | “These findings were supported by a large cohort study.” |
| Lifestyle Factor is used as a collective domain concept encompassing multiple lifestyle-related entity types in this schema, including Dietary Pattern, Nutrient, Physical Activity, Sleep Behavior, Stress & Mental State, and Social & Behavioral Context. | | | | | |

# 2 Literature retrieval and screening procedures.

## 2.1 Concept vocabulary construction

A controlled concept vocabulary was constructed based on MeSH terms and expert-reviewed supplementary terms to support standardized PubMed retrieval and knowledge graph construction. The vocabulary consisted of three categories: (1) lifestyle-related concepts (n=423), including diet, physical activity, sleep, stress, and other behavioral factors; (2) disease and health outcome concepts (n=182), including chronic diseases and related clinical outcomes; and (3) biomedical entity concepts (n=410), including mechanisms, nutrients, biomarkers, and other biological entities.

**Table S3.** Vocabulary categories

| Concept category | Description | Number of terms |
| --- | --- | --- |
| Lifestyle-related concepts | Dietary factors, physical activity, sleep, stress, and other behavioral factors | 423 |
| Disease and health outcome concepts | Chronic diseases, clinical outcomes, and related health conditions | 182 |
| Biomedical entity concepts | Biological mechanisms, nutrients, biomarkers, and other biomedical entities | 410 |

## 2.2 PubMed search strategy

PubMed retrieval was performed using a schema-driven concept vocabulary. Terms from the vocabulary were mapped to PubMed-compatible search terms, including MeSH terms and title/abstract keywords, and combined using Boolean operators to construct retrieval queries. The search query consisted of three concept blocks: (1) lifestyle-related concepts, (2) clinical outcome and disease-related concepts, and (3) biological mechanism-related concepts. Lifestyle concepts were used as primary anchors and were combined with clinical or mechanistic concepts using Boolean operators.

Example query:

(

"Diet"[Mesh] OR "Nutrition" OR "Physical Activity"

OR "Exercise" OR "Sleep" OR "Stress Management"

)

AND

(

"Chronic Disease"[Mesh]

OR

"Cardiovascular Diseases"[Mesh]

OR

"Diabetes Mellitus"[Mesh]

)

## 2.3 Inclusion and exclusion criteria

**Table S4.** Inclusion criteria

| **Criterion** | **Description** |
| --- | --- |
| Database | PubMed indexed publications |
| Time period | January 2014–December 2024 |
| Study population | Human-related evidence |
| Content relevance | Lifestyle factors, mechanisms, diseases, or management-related evidence |
| Data availability | Abstract available for extraction |

**Table S5.** Exclusion criteria

| **Criterion** | **Description** |
| --- | --- |
| Publication type | Non-research records without relevant evidence. |
| Review | Narrative or systematic reviews without original data. |
| Species | Animal-only studies |
| Time | Published before January 2014 |
| Content | Irrelevant lifestyle or disease topics |
| Abstract | Abstract length <50 characters |
| Duplicate | Duplicate records |

## 2.4 Composition of the 170,000-abstract corpus by study design

**Table S6.** Composition of study design

| **PubMed publication type** | **Number** | **Percentage** | **Normalized weight** |
| --- | --- | --- | --- |
| Meta-analysis | 13017 | 7.65% | 0.95 |
| Randomized Controlled Trial | 45648 | 26.81% | 0.90 |
| Cohort Study | 44875 | 26.36% | 0.75 |
| Case-Control Study | 19191 | 11.27% | 0.60 |
| Cross-Sectional Study | 33267 | 19.54% | 0.45 |
| Case Report / Expert Opinion / others | 14270 | 8.38% | 0.30 |

**Note:** *Records without a PubMed Publication Type (1.53%) were assigned the default weight of 0.30 and included in the 'Case Report / Expert Opinion / others' category.* *The mapped proportions reflect the retrieved corpus and do not necessarily generalise to the entire lifestyle literature.*

# 3 Prompt for knowledge extracted

**System Role Prompt**

You are a biomedical text mining expert specialized in lifestyle medicine and chronic disease management.Your task is to extract structured knowledge from biomedical abstracts to support evidence-based healthy lifestyle management and knowledge graph construction.

**Task Definition**

Given a biomedical abstract, perform the following two tasks under a predefined schema:

(1) Entity Extraction

Identify and classify text spans into one of the predefined entity types.

(2) Relation Extraction

Identify valid semantic relations between extracted entities according to the predefined relation set.

Only relations explicitly supported by the abstract content should be extracted. Do not infer unstated associations.

**Input:**

Schema definitions with examples: Predefined Entity Types (13 categories), Predefined Relation Types (14 categories)

Input text {"abstract"}

**Annotation Guidelines**

To ensure consistency and compatibility with downstream knowledge graph construction, apply the following rules:

1. Non-overlapping entities: Extract the minimal complete semantic unit without overlapping spans.
2. Non-nested entities: Avoid nested entity annotations.
3. Minimal punctuation: Remove trailing punctuation marks from entity spans.
4. Explicit grounding: Extract only relations that are explicitly stated or directly supported by the abstract.
5. No speculative inference: Do not generate relations based solely on general domain knowledge.

**Output:**

#Output with JSON

Entity Output：{entity name, entity type}，

Relation Output：{entity 1, entity type 1, relationship, entity 2, entity type 2}

# 4 Prompt for rule-guided triple validation and correction

**System Role Prompt**

You are a domain-aware knowledge graph verifier for lifestyle and biomedical knowledge. Your task is to validate and, if necessary, correct triples generated from biomedical abstracts according to a predefined schema and evidence-based constraints.

**Input:**
A set of extracted triples in JSON format, together with the predefined entity types, relation types, and schema examples.

**Task Instructions:**
Please perform the following steps sequentially for each group of generated triples:

1. Quantity Check

For each relation type, check whether the number of generated triples meets the minimum required threshold (default = 3).

If the number of triples for a given relation type is below the threshold, label the output as “Quantity insufficient”, indicating inadequate relational coverage for reliable knowledge integration.

2. Format and Structural Consistency Check

Verify whether each triple strictly conforms to the predefined schema and example format.

Classify errors as follows:

- Format error: the triple does not match the required JSON or schema structure.
- Head entity error: the head entity type does not match the predefined schema.
- Head–tail contradiction: the head entity and tail entity are identical or semantically incompatible.

3. Evidence support check

Examine whether the asserted relation in each triple is explicitly supported by the content of the source abstract. If the relationship cannot be directly inferred from the described study findings, or if the involved entities are mentioned independently without an explicit relational statement, the triple should be labeled as “Insufficient evidence.”

**Error Correction:**
For any detected error, regenerate the affected triple(s) by strictly following the predefined schema, entity types, and relation definitions, while ensuring logical consistency with evidence-based lifestyle knowledge.
Multiple correction attempts are allowed. Select the final output only if it satisfies schema compliance, passes all checks above.

**Output:**
A corrected set of triples in standardized JSON format, accompanied by error type labels for any modifications performed.

# 5 Descriptive statistics of EW-LKG: entity count per type, triplet count per semantic relation.

**Table S7.** Statistics of EW-LKG

| Knowledge Graph | Results (n, %) |
| --- | --- |
| Entity per type (n=155,986) | |
| Disease | 18420,11.81% |
| Health Outcome | 12365,7.93% |
| Intermediate Mechanism | 21740,13.94% |
| Population | 9865,6.32% |
| Dietary Pattern | 8940,5.73% |
| Nutrient | 24180,15.50% |
| Physical Activity | 7460,4.78% |
| Sleep Behavior | 6125,3.93% |
| Stress & Mental State | 7310,4.69% |
| Social & Behavioral Context | 6580,4.22% |
| Clinical Manifestation | 13420,8.60% |
| Intervention | 10365,6.64% |
| Drug | 9216,5.91% |
| Triplets per semantic relation (n=201,969) | |
| <Any Lifestyle Factor, associated_with, Disease / Health Outcome> | 48711,24.12% |
| <Lifestyle Factor / Nutrient, increases_risk_of, Disease / Health Outcome> | 28432,14.08% |
| <Lifestyle Factor / Intervention, reduces_risk_of, Disease / Health Outcome> | 22071,10.93% |
| <Lifestyle Factor / Nutrient, affects_mechanism, Intermediate Mechanism> | 24815,12.29% |
| <Lifestyle Factor, mediated_by, Intermediate Mechanism> | 11523,5.71% |
| <Intermediate Mechanism, contributes_to, Disease> | 14771,7.31% |
| <Disease, leads_to, Clinical Manifestation / Health Outcome> | 12637,6.26% |
| <Disease, has_clinical_manifestation, Clinical Manifestation> | 8951,4.43% |
| <Intervention / Lifestyle Factor, recommended_for, Population> | 7172,3.55% |
| <Disease, high_risk_population, Population> | 6319,3.13% |
| <Drug / Intervention, targets, Disease / Intermediate Mechanism> | 9472,4.69% |
| <Intervention / Lifestyle Factor, improves_outcome_of, Health Outcome> | 5981,2.96% |
| <Lifestyle Factor, worsens_outcome_of, Health Outcome> | 1114,0.55% |

# 6 EW-LKG community detection and validation

**Meta-path Projection.** To accommodate the heterogeneous, multi-relational nature of EW-LKG, we performed meta-path projection prior to community detection. Three clinically justified meta-paths were defined based on the EW-LKG schema:

1. **Lifestyle → Disease (direct):**

(PhysicalActivity | DietaryPattern | Nutrient | SleepBehavior | StressMentalState | SocialBehavioralContext) –[associated_with | increases_risk_of | reduces_risk_of]→ (Disease | HealthOutcome)

2. **Lifestyle → Mechanism → Disease (mediated):**

(Lifestyle) –[affects_mechanism]→ (IntermediateMechanism) –[contributes_to]→ (Disease | HealthOutcome)

3. **Lifestyle / Intervention → Population (contextual):**

(Lifestyle | Intervention) –[recommended_for | high_risk_population]→ (Population)

For each meta-path, we enumerated all path instances in EW-LKG using Neo4j Cypher queries. Node pairs connected by at least one path instance were linked in a projected homogeneous graph, with edge weights equal to the aggregated count of path instances across all three meta-paths.

**Community Detection.** The projected graph was imported into Python (igraph 0.10.x, leidenalg 0.10.x). The Leiden algorithm was applied using the RBConfigurationVertexPartition objective with resolution parameter γ = 1.0 and edge weights. This yielded 312 communities.

**Validation.**

*Entity-type purity.* For each community, we identified the most frequent entity type (among the 13 predefined schema classes) and calculated its proportion within that community. The mean purity across all 312 communities was 0.73 (SD 0.18).

*Blinded expert assessment of community coherence.* Five communities were randomly sampled, stratified by size (one small <50 entities, two medium 50–500, two large >500). Within each sampled community, five representative nodes were selected: the three highest-degree nodes and two additional nodes chosen by uniform random sampling. The resulting 25 nodes (grouped by community) were presented to three domain experts (chronic disease management specialist, clinical nutrition specialist, lifestyle medicine researcher) in randomized order with community labels and algorithmic origin concealed. Experts independently rated the topical coherence of each community's node set on a 5-point Likert scale (1 = nodes are semantically unrelated; 5 = nodes clearly belong to a single recognizable clinical or biological theme). The mean score was 4.2 (SD 0.5).

**Results Summary.** Modularity = 0.64; mean community size = 499 (SD 1,240); 28 communities contained >500 entities.

# 7 Prompt templates for the three QA configurations

Prompt 1. Baseline Retrieval-Augmented Generation (RAG)

You are a clinical assistant specialized in chronic disease lifestyle management.

Answer the following question based only on the retrieved biomedical literature provided below.

Instructions:
- Generate a concise and evidence-based answer.
- Do not use external knowledge.
- If the retrieved literature is insufficient, state that the evidence is insufficient.
- Cite the supporting literature where appropriate.

Question:
{question}

Retrieved abstracts (Top-5):
{retrieved_documents}

Please provide:

1. A concise answer.

2. Cite the corresponding PMID.

Answer:

Prompt 2. Knowledge Graph–Guided Prompting

You are a clinical assistant specializing in chronic disease lifestyle management.

Answer the question based ONLY on the structured knowledge retrieved from the evidence-weighted lifestyle knowledge graph.

Question:

{Question}

Recognized entities:

{Entities}

Retrieved knowledge:

{Triples with PMID}

Please provide:

1. A concise answer.

2. Explain the supporting relationships.

3. Cite the corresponding PMID.

Answer:

Prompt 3. Path-Aware Prompting

You are a clinical assistant specializing in chronic disease lifestyle management.

Answer the question based ONLY on the provided reasoning paths.

Question:

{Question}

Recognized entities:

{Entities}

Top-3 reasoning paths ranked by PathScore:

{Reasoning Paths}

Evidence sources:

{PMID}

Please provide:

1. A concise answer.

2. Explain the reasoning process based on the provided paths.

3. Cite the supporting PMID.

Answer:

# 8 Cypher templates

**Single-entity path retrieval.**

Given a query entity, the system enumerates all valid reasoning paths of 1–3 hops emanating from that entity. Only relations with confidence scores above a predefined threshold are traversed. Each candidate path is scored by the geometric mean of its constituent relation confidences, and the top-*k* highest-scoring paths are returned as structured evidence chains.

//	$entity_id	: String

//	$max_hops	: Integer default 3

//	$min_confidence	: Float	default 0.3

//	$top_k	: Integer default 3

//

// PathScore：

//	(c1 × c2 × ... × cn) ^ (1/n)

// ============================================================

MATCH path = (e:Entity {mesh_id: $entity_id})-[rels*1..$max_hops]->(n)

WHERE

n <> e

AND ALL(r IN rels WHERE r.confidence >= $min_confidence)

WITH

e,

n,

path,

rels,

length(path) AS hops,

reduce(

product = 1.0,

r IN rels |

product * r.confidence

) ^ (1.0 / length(path)) AS pathScore

RETURN

e.name AS sourceEntity,

e.mesh_id AS sourceMeshId,

[

node IN nodes(path) |

{

name: node.name,

meshId: node.mesh_id,

type: labels(node)[0]

}

] AS pathNodes,

[

r IN rels |

{

relationType: type(r),

confidence: r.confidence,

pmid: r.pmid

}

] AS evidenceChain,

n.name AS endpointEntity,

n.mesh_id AS endpointMeshId,

hops AS hopCount,

pathScore AS pathConfidence

ORDER BY

pathConfidence DESC,

hopCount ASC

LIMIT $top_k

**Multi-entity association retrieval.**

When multiple entities are identified in a query, the system constructs a pairwise association network by searching for 1–3 hop paths between every unique entity pair. Paths are filtered by a minimum per-edge confidence threshold and ranked globally by the same geometric-mean PathScore across all entity pairs. The top-k paths from the entire candidate pool are selected to provide interconnected, multi-perspective evidence for the query.

// $entity_ids	: List[String]

//	$max_hops	: Integer default 3

//	$min_confidence	: Float	default 0.4

//	$top_k_global	: Integer default 3

//

// PathScore：

//	(c1 × c2 × ... × cn) ^ (1/n)

// ============================================================

MATCH (src:Entity)

WHERE src.mesh_id IN $entity_ids

MATCH (tgt:Entity)

WHERE tgt.mesh_id IN $entity_ids

AND id(src) < id(tgt)

MATCH path = (src)-[rels*1..$max_hops]->(tgt)

WHERE

ALL(r IN rels WHERE r.confidence >= $min_confidence)

WITH

src,

tgt,

path,

rels,

length(path) AS hops,

reduce(

product = 1.0,

r IN rels |

product * r.confidence

) ^ (1.0 / length(path)) AS pathScore

RETURN

src.name AS fromEntity,

src.mesh_id AS fromMeshId,

labels(src)[0] AS fromType,

tgt.name AS toEntity,

tgt.mesh_id AS toMeshId,

labels(tgt)[0] AS toType,

[

node IN nodes(path) |

{

name: node.name,

meshId: node.mesh_id,

type: labels(node)[0]

}

] AS pathNodes,

[

r IN rels |

{

relationType: type(r),

confidence: r.confidence,

pmid: r.pmid

}

] AS evidenceChain,

hops AS hopCount,

pathScore AS pathConfidence

ORDER BY

pathConfidence DESC,

hopCount ASC

LIMIT $top_k_global

# 9 Sensitivity of overall QA accuracy to alternative evidence weighting schemes

**Table S8.** QA accuracy to alternative evidence weighting schemes

| **Configuration** | **Overall Accuracy** |
| --- | --- |
| Baseline RAG | 0.733 |
| Path-selection (OCEBM, geometric mean) | 0.894 |
| Path-selection (Equal weight) | 0.883 |
| Path-selection (shortest-path selection) | 0.777 |
| Path-selection (random-selection) | 0.791 |

Equal weight = 0.5 for all study designs.

# 10 Validation of Evidence-Weighted Confidence Stratification in the EW-LKG

**Table S9. Supporting Publication Counts Across Confidence Strata**

| **Confidence Stratum** | **Relations, n (%)** | **Single-support Relations, n (%)** | **Supporting Publications, n** | **Mean Publications per Relation** |
| --- | --- | --- | --- | --- |
| Low (<0.60) | 39,953  (19.78%) | 34,713  (86.88%) | 45,129 | 1.13 |
| Medium (0.60–0.80) | 104,213  (51.60%) | 40,506  (38.87%) | 182,484 | 1.75 |
| High (>0.80) | 57,803  (28.62%) | 4,561  (7.89%) | 182,943 | 3.16 |

Table S9 summarizes the number of supporting publications associated with relations across the three confidence strata. Most low-confidence relations (86.9%) were supported by a single publication, whereas single-support relations accounted for only 7.9% of high-confidence relations. The mean number of supporting publications increased from 1.13 in the low-confidence stratum to 3.16 in the high-confidence stratum. These results show that high-confidence relations were generally supported by multiple publications rather than being predominantly determined by a single source.

**Table S10. Study Design Composition of Supporting Publications Across Confidence Strata**

| **Study Design** | **Low (<0.60), n (%)** | **Medium (0.60–0.80), n (%)** | **High (>0.80), n (%)** |
| --- | --- | --- | --- |
| Meta-analysis | 0 (0%) | 0 (0%) | 19,728 (10.8%) |
| Randomized controlled trial (RCT) | 0 (0%) | 0 (0%) | 49,608 (27.1%) |
| Cohort study | 0 (0%) | 35,971 (19.7%) | 47,034(25.7%) |
| Case-control study | 0 (0%) | 43,291 (23.7%) | 23,133 (12.6%) |
| Cross-sectional study | 29,573 (65.5%) | 74,699 (40.9%) | 31,869 (17.4%) |
| Case report | 15,556(34.5%) | 28,523 (15.6%) | 11,571 (6.3%) |

Table S10 further characterizes the study-design composition of the supporting evidence within each confidence stratum. Importantly, no meta-analyses or RCTs were assigned to the low- or medium-confidence strata. Low-confidence relations were supported exclusively by cross-sectional studies and case reports, whereas medium-confidence relations were supported by cohort, case-control, cross-sectional, and case-report evidence. Higher-level evidence, including meta-analyses and RCTs, occurred only in the high-confidence stratum.

**Table S11. Study-Design Composition of High-Confidence Relations**

| Composition of Supporting Evidence | Confidence | Relations, n (%) |
| --- | --- | --- |
| At least one meta-analysis or RCT | ≥0.90 | 41,753(72.2%) |
| Observational/low-level evidence accumulation, subtotal |  | 16,050(27.8%) |
| — Two cohort studies | 0.9375 | 4,513 |
| — Cohort + case-control | 0.9000 | 3,471 |
| — Cohort + cross-sectional study | 0.8625 | 2,173 |
| — Two case-control studies | 0.8400 | 1,971 |
| — Cohort + case report | 0.8250 | 1,168 |
| — Three cross-sectional studies | 0.8336 | 1,296 |
| — Other combinations* |  | 1,458 |

*Other combinations include combinations of lower-weight evidence that yield a confidence score above 0.80.

To further examine the potential for evidence accumulation to produce high confidence in the absence of high-level evidence, we analyzed the composition of all 57,803 high-confidence relations (Supplementary Table S11). A total of 41,753 relations (72.2%) contained at least one meta-analysis or RCT. The remaining 16,050 relations (27.8%) achieved high confidence through combinations of observational or lower-level evidence. Several such combinations can indeed exceed the 0.80 threshold, for example, two cohort studies (0.9375) or a cohort study combined with a case-control study (0.9000). Thus, the analysis suggests that evidence accumulation is possible under the proposed formulation, but also shows that lower-level evidence accumulation accounts for a minority rather than the majority of high-confidence relations in the current graph.

# 11 Stratified PathScore statistics by hop count and question category.

(1) **Stratification by hop count.** Table S12 reports PathScore means and SDs within each hop-count stratum under the revised geometric-mean formulation. Correct responses consistently scored higher than incorrect responses across all hop counts:

**Table S12. PathScore–correctness association stratified by question type (hop level)**

| **Question type**  **(hop level)** | **N (Correct)** | **PathScore (Correct)** | **N (Incorrect)** | **PathScore (Incorrect)** | ***p*-value** |
| --- | --- | --- | --- | --- | --- |
| 1-hop | 184 | 0.83 ± 0.08 | 16 | 0.64 ± 0.06 | 3.49× 10⁻10 |
| 2-hop | 208 | 0.79 ± 0.09 | 28 | 0.61 ± 0.07 | 3.52 × 10⁻¹5 |
| ≥3-hop | 74 | 0.74 ± 0.07 | 11 | 0.59 ± 0.05 | 1.69 × 10⁻⁷ |
| **Overall** | **466** | **0.80 ±0.089** | **55** | **0.62 ± 0.065** | **3.23 × 10⁻^30^** |

The positive association between higher PathScores and correctness holds within every stratum, indicating that the effect is not an artefact of shorter paths being easier to answer.

(2) **Stratification by question category.** The association also replicates across all four question categories:

**Table S13. PathScore–correctness association stratified by question category**

| **Question Category** | **N (Correct)** | **PathScore (Correct)** | **N (Incorrect)** | **PathScore (Incorrect)** | ***p*-value** |
| --- | --- | --- | --- | --- | --- |
| Lifestyle Intervention and Management | 135 | 0.80 ± 0.09 | 16 | 0.63 ± 0.07 | 1.48 × 10⁻8 |
| Mechanism-Informed Explanation | 120 | 0.78 ± 0.09 | 17 | 0.58 ± 0.06 | 2.64 × 10⁻12 |
| Population-Specific Lifestyle Guidance | 87 | 0.80 ± 0.08 | 11 | 0.63 ± 0.06 | 6.80 × 10⁻7 |
| Risk and Association Assessment | 124 | 0.80 ± 0.08 | 11 | 0.64 ± 0.07 | 1.12 × 10⁻5 |
| **Overall** | **466** | **0.80 ±0.089** | 55 | **0.62 ± 0.065** | **3.23 × 10⁻^30^** |

# 12 Confusion matrices: Path-selection vs. Baseline RAG/KG-guided/ KRAGEN

**Table S14. Path-selection vs. Baseline RAG**

|  |  | Both correct | baseline RAG correct, Path wrong | baseline RAG wrong, Path correct | Both wrong | Total | McNemar P |
| --- | --- | --- | --- | --- | --- | --- | --- |
| By model | |  |  |  |  |  |  |
|  | Qwen3-Max | 310 | 11 | 107 | 93 | 521 | < 0.001 |
|  | GPT-5.2 | 367 | 9 | 92 | 53 | 521 | < 0.001 |
|  | LLaMA-4-Maverick | 301 | 11 | 94 | 115 | 521 | < 0.001 |
|  | DeepSeek V3.2-Exp | 369 | 13 | 97 | 42 | 521 | < 0.001 |
| By reasoning complexity | | |  |  |  |  |  |
|  | Direct (1-hop) | 153 | 5 | 31 | 11 | 200 | < 0.001 |
|  | Mechanism-mediated (2-hop) | 162 | 5 | 46 | 23 | 236 | < 0.001 |
|  | Multi-factor (≥3 hops) | 54 | 3 | 20 | 8 | 85 | < 0.001 |

**Table S15.Path-selection vs. KG-guided**

|  |  | Both correct | KG correct, Path wrong | KG wrong, Path correct | Both wrong | Total | McNemar P |
| --- | --- | --- | --- | --- | --- | --- | --- |
| By model | |  |  |  |  |  |  |
|  | Qwen3-Max | 338 | 11 | 79 | 93 | 521 | < 0.001 |
|  | GPT-5.2 | 415 | 7 | 44 | 55 | 521 | < 0.001 |
|  | LLaMA-4-Maverick | 352 | 5 | 43 | 121 | 521 | < 0.001 |
|  | DeepSeek V3.2-Exp | 414 | 8 | 52 | 47 | 521 | < 0.001 |
| By reasoning complexity | | |  |  |  |  |  |
|  | Direct (1-hop) | 165 | 4 | 19 | 12 | 200 | < 0.05 |
|  | Mechanism-mediated (2-hop) | 188 | 2 | 20 | 26 | 236 | < 0.05 |
|  | Multi-factor (≥3 hops) | 61 | 2 | 13 | 9 | 85 | < 0.05 |

**Table S16. Path-selection vs. KRAGEN**

|  |  | Both correct | KRAGEN correct, Path wrong | KRAGEN wrong, Path correct | Both wrong | Total | McNemar P |
| --- | --- | --- | --- | --- | --- | --- | --- |
|  | Qwen3-Max | 384 | 9 | 33 | 95 | 521 | < 0.001 |
|  | GPT-5.2 | 443 | 5 | 16 | 57 | 521 | 0.029 |
|  | LLaMA-4-Maverick | 364 | 7 | 31 | 119 | 521 | < 0.001 |
|  | DeepSeek V3.2-Exp | 435 | 6 | 31 | 49 | 521 | < 0.001 |

**Table S17. Path-selection vs. no-retrieval**

|  |  | Both correct | no-retrieval correct, Path wrong | no-retrieval wrong, Path correct | Both wrong | Total | McNemar P |
| --- | --- | --- | --- | --- | --- | --- | --- |
|  | Qwen3-Max | 207 | 8 | 210 | 96 | 521 | < 0.001 |
|  | GPT-5.2 | 236 | 4 | 223 | 58 | 521 | < 0.001 |
|  | LLaMA-4-Maverick | 208 | 6 | 187 | 120 | 521 | < 0.001 |
|  | DeepSeek V3.2-Exp | 239 | 5 | 227 | 50 | 521 | < 0.001 |

# 13 Repeat-Run Agreement for Extraction at T=0.7

To assess the consistency of our extraction pipeline at T=0.7, we conducted three repeated extraction runs on the 200-abstract evaluation set using identical prompts and model version (DeepSeek V3.2-Exp). Results:

**Table S18. Repeat-Run Agreement at T=0.7**

| Metric | Run 1 | Run 2 | Run 3 |
| --- | --- | --- | --- |
| Total triples extracted | 631 | 624 | 638 |
| Triples matching gold standard | 557 | 548 | 561 |
| **Precision** | 88.2% | 87.8% | 87.9% |
| **Recall** | 90.0% | 88.5% | 90.6% |
| **F1-score** | 89.1% | 88.2% | 89.3% |

A chi‑square test of homogeneity showed no statistically significant difference in the proportions of correct extractions among the three runs (χ² = 0.066, df=2, p = 0.967).
